# The Role of Chromosomal Instability and Epigenetics in Colorectal Cancers Lacking *β*-Catenin/TCF Regulated Transcription

**DOI:** 10.1155/2016/6089658

**Published:** 2016-03-07

**Authors:** Wael M. Abdel-Rahman, Johanna E. Lotsari-Salomaa, Sippy Kaur, Anni Niskakoski, Sakari Knuutila, Heikki Järvinen, Jukka-Pekka Mecklin, Päivi Peltomäki

**Affiliations:** ^1^Department of Medical Laboratory Sciences, College of Health Sciences and Sharjah Institute for Medical Research (SIMR), University of Sharjah, P.O. Box 27272, Sharjah, UAE; ^2^Department of Medical and Clinical Genetics, University of Helsinki, 00290 Helsinki, Finland; ^3^Department of Pathology, Haartman Institute and HUSLAB, University of Helsinki and Helsinki University Central Hospital, Helsinki, 00029 HUS, Finland; ^4^Second Department of Surgery, Helsinki University Central Hospital, Helsinki, 00029 HUS, Finland; ^5^Department of Surgery, Jyväskylä Central Hospital, 40620 Jyväskylä, Finland; ^6^Institute of Clinical Medicine, University of Eastern Finland, Kuopio, Finland

## Abstract

All colorectal cancer cell lines except RKO displayed active *β*-catenin/TCF regulated transcription. This feature of RKO was noted in familial colon cancers; hence our aim was to dissect its carcinogenic mechanism. MFISH and CGH revealed distinct instability of chromosome structure in RKO. Gene expression microarray of RKO versus 7 colon cancer lines (with active Wnt signaling) and 3 normal specimens revealed 611 differentially expressed genes. The majority of the tested gene loci were susceptible to LOH in primary tumors with various *β*-catenin localizations as a surrogate marker for *β*-catenin activation. The immunohistochemistry of selected genes (*IFI16*,* RGS4*,* MCTP1*,* DGKI*,* OBCAM*/*OPCML*, and* GLIPR1*) confirmed that they were differentially expressed in clinical specimens. Since epigenetic mechanisms can contribute to expression changes, selected target genes were evaluated for promoter methylation in patient specimens from sporadic and hereditary colorectal cancers.* CMTM3*,* DGKI*, and* OPCML* were frequently hypermethylated in both groups, whereas* KLK10*,* EPCAM*, and* DLC1* displayed subgroup specificity. The overall fraction of hypermethylated genes was higher in tumors with membranous *β*-catenin. We identified novel genes in colorectal carcinogenesis that might be useful in personalized tumor profiling. Tumors with inactive Wnt signaling are a heterogeneous group displaying interaction of chromosomal instability, Wnt signaling, and epigenetics.

## 1. Introduction

Early research into the role of* APC* mutations in colorectal carcinogenesis showed that the majority of colorectal cancer cell lines have acquired activated *β*-catenin/TCF regulated transcription and hence active canonical Wnt signaling pathway, as a result of inactivation of APC or activating mutations of *β*-catenin gene* CTNNB1* [1]. This finding was confirmed in further studies on cell lines and primary tumors [2, 3]. The Cancer Genome Atlas Research Network showed that 93% of colorectal cancers had mutations in this pathway; most often, these changes appeared in the* APC* tumor suppressor gene [4]. Exceptionally, the cell line RKO did not have mutations in* APC* or* CTNNB1* and did not show any evidence of activated *β*-catenin/TCF regulated transcription [2, 5]. This finding was of interest to cancer researchers and was confirmed in separate study through robust functional analyses [5]. In primary colorectal tumors, immunohistochemical detection of *β*-catenin subcellular localization was established as a surrogate marker for its activity, where nuclear *β*-catenin localization indicated activated *β*-catenin/TCF regulated transcription and membranous localization of *β*-catenin indicated inactive *β*-catenin/TCF regulated transcription. Using this approach, the majority of primary colon tumors showed nuclear *β*-catenin with the exception of few subsets including familial colon cancer [6]. Hence, investigation of carcinogenic mechanisms in such category might uncover novel pathways in colorectal carcinogenesis.

Extensive studies showed that genetic instability and epigenetic changes play a significant role in colorectal carcinogenesis. Different patterns of genetic instability were described in colorectal cancers including chromosomal instability which may have diverse underlying causes and microsatellite instability (MSI) due to deficiency in the mismatch repair (MMR) system [7]. Chromosomal instability could affect chromosomal number and/or structure [8] and the latter could manifest as gross structural rearrangements and/or subtle changes resulting in loss of heterozygosity (LOH). Further studies into the mechanisms of LOH development showed that environmental carcinogens have the ability to induce genome-wide LOH without persistent chromosomal instability [9]. Epigenetic alterations could also reflect the effect of the environmental toxins and/or ethnicity [10]. Interestingly, chromosomal instability, MSI, and epigenetics were shown to be independent prognostic factor in stage II/III colorectal cancer [11, 12]. Therefore, to understand the carcinogenesis of cells lacking *β*-catenin/TCF regulated transcription, it is important to address the interaction of these mechanisms in such cells.

We compared the RKO cell lines to seven classical colorectal cancer cell lines (with active Wnt signaling) and three normal colon mucosa specimens. The seven cell lines were chosen to be MSI similar to RKO, to eliminate the influence of this mechanism.

## 2. Materials and Methods

### 2.1. Patients and Samples

The colon cancer cell lines were RKO, HCA7, KM12, LoVo, HCT15, HCT116, SW48, and LIM1215. RKO was the only cell line with no mutation in* APC* or* CTNNB1*. To maintain this as the differentiating factor, cell lines for microarray analysis were selected to be MSI similar to RKO and, moreover, the majority of these cells were CpG island methylator phenotype- (CIMP-) positive similar to RKO. Many of these features were confirmed in our lab (see Section 3). We included three, high purity normal colon RNA samples (from Ambion/Life Technologies) in the microarray analysis. The primary colorectal samples for methylation analysis consisted of 110 colorectal cancers from Finland, including 27 from Lynch syndrome families and 83 sporadic tumors [13, 14]. For immunohistochemistry, there were additional sections from 12 sporadic adenomas, 38 cancers from 11 Finnish Lynch syndrome families, and 19 specimens representing Familial Colorectal Cancer Type X (FCCX), hereditary nonpolyposis colorectal carcinoma without DNA mismatch repair defects. Tumors were examined by microsatellite instability testing and MMR protein expression studies by immunohistochemical staining [13]. For the LOH, a pilot study was performed on 22 MSS sporadic cancers (12 with nuclear and 10 with membranous *β*-catenin). Fresh frozen and/or paraffin derived specimens of tumor and matching normal tissues were collected from pathology departments of different hospitals and used for immunohistochemical analysis and DNA extraction according to standard protocols. The study was approved by the institutional review board of the Helsinki University Central Hospital (Dnro 466/E6/01) and the National Authority for Medicolegal Affairs (Dnro 1272/04/044/07).

### 2.2. APC,* CTNNB1*, and* BRAF* Mutations

APC mutation analysis was as described [15].* CTNNB1* exon 3 was directly sequenced as described [6].* BRAF* mutation analysis focused on codon 600 and was conducted as described in Niskakoski et al. [16].

### 2.3. M-FISH and CGH

Multiplex fluorescence in situ hybridization (M-FISH) was performed using the Xcyting colors (MetaSystems GmbH, Altlussheim, Germany) according to the manufacturer's protocol. Data were analysed using ISIS 4.4.21 software (MetaSystems GmbH). Comparative genomic hybridization (CGH) was performed and results were analyzed according standard protocols. Briefly, tumor DNA and reference DNA (genomic DNA from peripheral blood leukocytes from normal donors) were labelled by nick translation with fluorescein-iso-thiocyanate (FITC)-conjugated dCTP and dUTP (DuPont, Boston, MA, USA), and Texas Red-conjugated dCTP and dUTP (Dupont), respectively, to obtain fragments ranging from 600 to 2000 bp. The results were analyzed using the ISIS digital image analysis system (MetaSystems GmbH, Altlussheim, Germany), based on an integrated high-sensitivity monochrome charge-coupled device (CCD) camera and automated CGH analysis software.

### 2.4. Gene Expression Microarray

Gene expression was analyzed using Affymetrix Human Genome U133 Plus 2.0 GeneChip® microarrays (Affymetrix, Santa Clara, CA), containing over 54 000 probe sets covering 47 000 transcripts. The samples for microarray were biological duplicates of all the 8 cell lines plus the three normal mucosal specimens.

#### 2.4.1. Preparation of RNA

Total RNA was extracted and purified using Qiagen RNeasy kit (Qiagen Inc., Valencia, CA, USA). RNA integrity and yield were assessed by Agilent Bioanalyzer 2100 (Agilent Technologies, CA, USA). Only samples with an RNA integrity number (RIN) higher than 7.0 were included in the analysis.

#### 2.4.2. Hybridization and Image Analysis

Samples were amplified, labelled and hybridized according to manufacturer's protocol and as described in Nymark et al. [19]. The arrays were washed and stained with streptavidin-phycoerythrin in an Affymetrix GeneChip Fluidics station 450, and scanned with Affymetrix GeneChip Scanner 3000. The image was analyzed using the GeneChip operating software (GCOS; Affymetrix, Santa Clara, CA) and comparison analysis was done according to the instructions provided by the manufacturer. After image acquisition, raw fluorescent signal (cel. file) from Affymetrix GeneChip Operating Software (GCOS) was used for analysis.

Microarray data were analyzed using GeneSpring GX12.0 software and processed using robust multiarray analysis (RMA) algorithm for background correction, normalization and log2-transformation. Unpaired *t*-test analysis with Benjamini-Hochberg multiple testing correction was utilized to obtain genes whose fold change between Wnt inactive and Wnt active colorectal cancer cell lines was ≥2.0 (with *P* value cut-off of <0.05). Unsupervised hierarchical clustering analysis was performed in GeneSpring software on normalised data for differentially expressed genes for all samples. The euclidean distance metric was used, this calculates the standard sum of squared distance between two entities, with centroid linkage (distance between two clusters is calculated as the average distance between their respective centroids weighted by the size of the clusters). The raw microarray data are deposited at GEO with the accession number GSE58058.

Pathway analysis was performed at the Database for Annotation, Visualization and Integrated Discovery (DAVID) v6.7, NIAID, NIH at http://david.abcc.ncifcrf.gov/home.jsp using KEGG and Biocarta annotation.

### 2.5. MSI and LOH Analysis

MSI status was determined using the Bethesda panel (BAT25, BAT26, D5S346, D2S123, and D17S250; [20]). The 31 dinucleotide repeat markers used for LOH analyses (Supplementary Table S1, in Supplementary Material available online at http://dx.doi.org/10.1155/2016/6089658) had high allele number and heterozygosity frequency. Primer sequences and PCR amplification conditions were retrieved through NCBI website (http://www.ncbi.nlm.nih.gov/). The experimental procedure and data analysis were as described [6]. Tumors with two or more unstable markers, or BAT25 or BAT26 instability alone were considered as having high-degree microsatellite instability (MSI-H). LOH study was performed on 22 cancers from the sporadic MSS series (12 with nuclear and 10 with membranous *β*-catenin).

### 2.6. Immunohistochemistry

The primary antibodies are listed in Supplementary Table S2. Immunohistochemistry was performed as described before [21] on a pilot series of 22 carcinomas (12 with nuclear *β*-catenin and 10 with membranous *β*-catenin) and then was extended to include all available sections.

### 2.7. Methylation Analysis

Methylation analysis was conducted by Methylation-specific multiplex ligation-dependent probe amplification (MS-MLPA). In brief, promoter-associated CpG islands were examined for methylation by bisulphite sequencing with primers indicated in Supplementary Table S3-A and representative HhaI sites used for MS-MLPA probe design according to the protocol of MRC-Holland (Supplementary Table S3-B). Upon digestion of the sample DNA/probe hybrids with the methylation sensitive restriction enzyme HhaI, only samples with the HhaI site methylated will generate an amplification product. Synthetic MS-MLPA probes for the chosen genes were added to the P300 commercial test (SALSA MLPA P300-A1 Reference-2 kit, MRC-Holland, Amsterdam, the Netherlands) for control fragments. The DLC1 gene had two separate MS-MLPA probes, with the probe for DLC1 isoform 4 (DLC1-i4) designed based on a previous publication [22]. Methylation dosage ratios (*D*_*m*_ values) were calculated as described [23].

Custom MS-MLPA assays were validated against bisulfite sequencing by using normal tissue and cancer cell lines. The technical threshold for methylation detection by MS-MLPA was 0.15 for all loci since *D*_*m*_ <0.15 by MS-MLPA correlated with the lack of methylation (T/T) by bisulphite sequencing, whereas *D*_*m*_ >0.15 correlated with methylation (C/T or C/C) by bisulphite sequencing. To evaluate increased methylation in tumor DNA versus normal DNA (i.e., hypermethylation), a normal tissue-based hypermethylation threshold was determined individually for each locus. The hypermethylation threshold was defined as the average *D*_*m*_ +1 standard deviation and derived from the combined set of normal mucosa specimens (*n* = 110) from all patient groups (Supplementary Table S4).

### 2.8. Statistical Analysis

Statistical analyses were performed using SPSS Statistics Software (IBM SPSS, Inc., Chicago, IL, USA). The percentages of tumors with hypermethylation were compared by Fisher Exact Probability Test from VassarStats Web site (http://www.vassarstats.net/tab2x2.html). Independent samples *t*-test was used for comparisons of two normally distributed tumor groups. One-way ANOVA was used for statistical analyses of normally distributed data. When methylation patterns between normal and tumor samples were examined parametric *t*-test for paired samples or nonparametric paired-samples Wilcoxon signed rank test was used. *P* values <0.05 (two-tailed) were considered significant.

## 3. Results

### 3.1. MSI,* APC*, and* CTNNB1* Status Showed That RKO Is Unique in Having Nonmutant* APC*/*CTNNB1*

RKO was the only cell line with no mutation in* APC or CTNNB1*. To maintain this as the a differentiating factor, cell lines for microarray analysis were selected to be MSI similar to RKO and the majority of these cells were CpG island methylator phenotype- (CIMP-) positive similar to RKO as well. We considered it important that these cell lines used for comparison shared these characteristics. The MSI status and the *β*-catenin gene (*CTNNB1*) mutation status were confirmed in our lab during previous studies [13, 24]. The* APC* gene was fully sequenced in the 3 cell lines HCA7, KM12, and RKO in our laboratory [24]. We found no mutations in RKO whereas frame shift mutations due to insertions of one nucleotide in exon 15 were present in the HCA7 and KM12 cell lines. The KM12 mutation was “c.5454_5455insA” and the HCA7 mutation was “c.4667_4668insA” as compared to GenBank accession number RefSeqM74088.1 (Supplementary Table S5).

Since RKO was the among the only in vitro colorectal cancer model holding* BRAF* mutation; we also extended the* BRAF* mutation analysis to the primary tumors. We found that* BRAF* V600E mutation was present as follows: sporadic MSI-colorectal cancers 17/33 (51%) (result was not available for 7), sporadic MSS- colorectal cancers 0/40 (0%) (result was not available for 3), and Lynch syndrome-colorectal cancers 0/15 (0%) (result was not available for 12).

### 3.2. M-FISH and CGH Profile of RKO Are Unique

**Figure 1:**
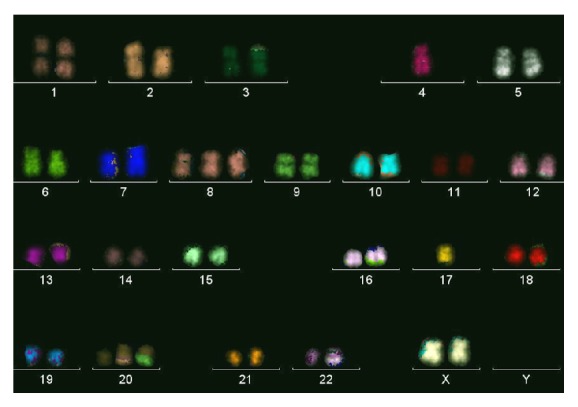
A representative metaphase from RKO cell line. Note that rearranged chromosomes described in Section 3 and not seen here were observed in two or more other metaphases.

The RKO karyotype by M-FISH (Figure 1) showed marked metaphase-to-metaphase heterogeneity. The karyotype of the most common clone (14/20 metaphases) was 47 (44~49), XX, del(2)(p21), der(3)t(3;5), del(5q), dup(7)(q21q36), +der(8)t(8;8)(p12;q21), del(9), +der(12)t(2;12), +der(20)t(9;20)(q22;p13), and der(22)t(16;22). In addition, there were some structural rearrangements present in two or more of the metaphases such as del(X), del(6p), der(12)t(5;12), der(10)t(2;10)(p21;q11), and der(20)t(2;20). To identify the nature of chromosomal instability and to allow comparison with the published karyotype of other colon cancer cell lines, variety of indices were calculated and compared to classical MSI and MSS cell lines from the published literature (Table 1). The total “aneuploid index” [17] equals the sum of all the nondisjunctional events required to generate the aberrant chromosome complement in a given cancer cell line. As shown in Table 1, the RKO cell line has a stable aneuploid index typical of the MSI cell lines [17]. The variability of centromere numbers [8] showed that RKO also had no numerical chromosomal instability similar to the MSI cell lines (Table 1). The number of rearranged chromosomes was used as a measure of instability of chromosome structure. RKO cell line had 14 rearranged chromosomes that were noted in two or more metaphases. This number was rather typical of the MSS cell lines. The CGH showed 9 copy number changes in RKO which were rev ish enh(7q21qter, 8q11qter, 9q22qter, 12pterq14, 16q22qter, 20, 22), amp(8q22qter, 9q34qter). This number was also closer to the MSS cells. Hence, both the M-FISH and CGH independently revealed instability of chromosome structure in RKO.

Karyotypes of other cell lines used here were available in previous publication by our group [8] or others [25, 26] except for LIM1215 which was karyotyped here for the first time by a molecular cytogenetic technique and it showed a stable karyotype, 46 (45–47), XY, del(3p), and der(13)t(1;13), consistent with being MSI line.

### 3.3. Expression Microarray Revealed Many Interesting Candidate Genes

**Figure 2:**
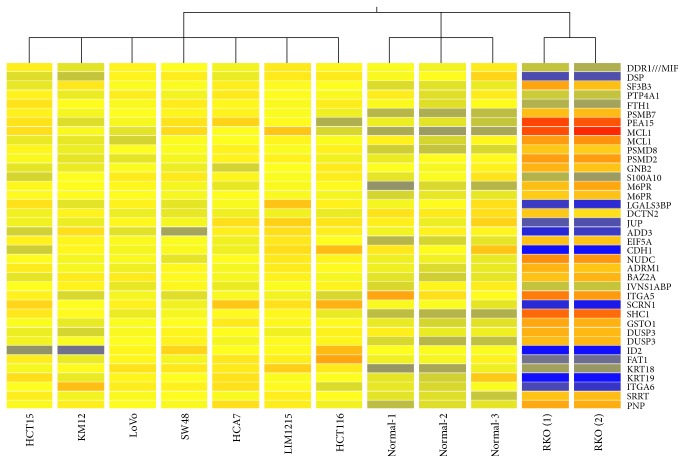
Cluster analysis based on the cDNA expression profiling of all colorectal cancer cell lines and normal colon specimens. The analysis was performed on GeneSpring GX software, version 11.0.2, Agilent Technologies.

In order to discover global gene expression patterns characterizing the rare colon cancer subgroup lacking constitutive *β*-catenin/TCF regulated transcription, the RKO cell line cDNA expression profile was compared to 10 specimens: 7 MSI colon cancer lines and three normal colon mucosa samples as described above. Taking a twofold change as threshold limit, there were 815 differentially expressed Affymetrix probes (272 downregulated and 543 upregulated) in RKO compared to Wnt-active colon cancer cell lines. The probes corresponded to 611 unique genes (399 downregulated and 212 downregulated, Supplementary Table S6). Cluster analysis showed that samples clustered into 3 distinct groups with RKO duplicate samples in one group, the normal samples in one group, and all other cancer cell lines in one group (Figure 2). Pathway analysis of the differentially expressed genes revealed that at least 20 pathways were affected including insulin signaling, ErbB signaling, T cell receptor signaling pathway and apoptosis. Wnt signaling pathway itself was represented by 3 differentially expressed genes (CCND3, PPP3CA, and PPP3CC). For accurate identification of *β*-catenin targets we compared our list to recent literature of Wnt targets in colon cancer [27]. We identified 23 Wnt target genes from our list when compared to Herbst et al. list of 335 genes. These were reduced to 17 genes when compared to the most stringent list of 193 identified by Herbst et al. [27] after they compared their data to the previous literature. Our 17 genes were ABCC3, BCL10, BCL2L11, C19orf33, C4orf34, DUSP3, ELF3, GPRC5A, KLHL29, MYOF, NAV3, RBM47, RNF43, S100A10, TEX10, TJP2, and TNS3.

### 3.4. LOH of the Selected Target Genes Was Common

**Figure 3:**
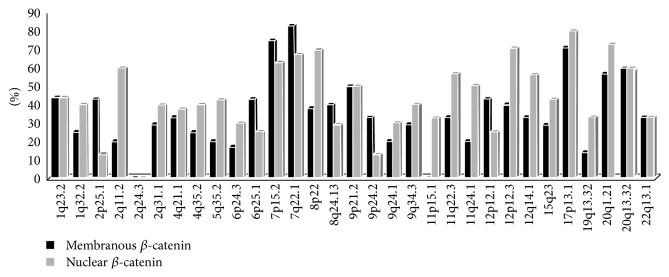
High frequency of LOH at all tested loci in 22 sporadic MSS colorectal cancers (12 with nuclear and 10 with membranous *β*-catenin) indicates significant role of the differentially expressed genes in carcinogenesis.

A total of 31 dinucleotide markers were analyzed for LOH in informative cases (i.e., heterozygous MSS cases) (Supplementary Table S1 and Figure 3). These markers were selected to be spread along the whole genome and to be close to differentially expressed genes from the microarrays. Both tumor categories, those with and without active Wnt signaling, were susceptible to LOH. All tested loci with the exception of 2q24.3 showed some degree of LOH in primary tumors. Overall, LOH frequency was slightly lower in tumors with inactive Wnt signaling but this did not reach statistical significance. Some of the loci such as D7S2564, D7S662, D8S552, D12S1728, D17S1353, D20S874, and D20S171 showed high overall frequency of LOH in more than 50% of the informative cases. 2p25.1 and 2q11.2 provided the best example of loci that were preferentially lost in one category (Figure 3). The results illustrated that many of the differentially expressed genes were also susceptible to LOH in primary tumors.

### 3.5. Immunohistochemistry Analysis of Target Genes

Genes with cancer-relevant function and with antibodies available were selected from the list of differentially expressed genes to be further evaluated by immunohistochemistry. The immunohistochemical staining results are shown in Table 2. Six genes selected for this analysis (*IFI16*,* RGS4*,* MCTP1*,* DGKI*,* OBCAM/OPCML*, and* GLIPR1*) showed variation of protein expression among the 22 tumors of the pilot study. For* RGS4*,* DGKI*,* OBCAM/OPCML*, and* GLIPR1* proteins, staining was extended to include all tumors available. Expression of* DGKI* and* GLIPR1* showed significant relationship with the *β*-catenin subcellular localization in a pattern similar to that observed in the microarray analysis of the cell lines (Table 2). Examples of the immunohistochemical staining are shown in supplementary Figure SF1.

### 3.6. Promoter Methylation of Target Genes Was Associated with *β*-Catenin Status

As promoter methylation can underlie expression changes, six genes from the cell line experiments, all with verified or putative roles in growth regulation and with CpG islands in the promoter region, were selected for methylation analyses by MS-MLPA. Four genes exhibited high expression in RKO compared to the other MSI colon cancer cell lines (*CMTM3*,* DGKI*,* OPCML*, and* DLC1*) whereas two genes had low gene expression in RKO (*EPCAM* and* KLK10*).* DLC1* showed a fold change <2 in the microarray analysis but it was included because of its potential significance as a tumor suppressor in colon and liver cancers [28, 29]. We found that the methylation status of these genes in the cell lines was generally consistent with the expression status from the microarray experiment (Supplementary Table S7).

Since the baseline levels of methylation may show locus and patient group-specific variation, thresholds for hypermethylation were determined as described in Section 2 and Supplementary Table S4 and used to compare tumors from the different patient groups (by Fisher's exact test). Three genes (*CMTM3*,* DGKI*, and* OPCML*) revealed high frequencies of hypermethylation (78–98%) and no apparent subgroup-specificity.* KLK10* and* EPCAM* showed Lynch syndrome-specific methylation patterns. For* KLK10*, 48% of Lynch colorectal tumors showed hypermethylation compared to 8% of sporadic MSI (*P* < 0.001) and 12% of sporadic MSS tumors (*P* < 0.001). Hypermethylation of* EPCAM* was restricted to Lynch syndrome colorectal cancers (30% versus 0% in the sporadic groups; *P* < 0.001).* DLC1* hypermethylation was characteristic of sporadic MSI colorectal cancer as compared to Lynch (28% versus 0% detected by the* DLC1* probe, *P* < 0.01) or sporadic MSS colorectal cancer (28% versus 5% detected by the* DLC1*_*i4* probe, *P* < 0.01).

To investigate the relationship between promoter hypermethylation and *β*-catenin subcellular localization, the number of gene promoters showing hypermethylation out of 7 (corresponding to the six genes examined, with two separate promoter regions,* DLC1* and* DLC1_i4*, for* DLC1*) was calculated for each tumor and compared with nuclear versus membranous localization of *β*-catenin. In all three patient groups (Lynch syndrome, sporadic MSI, and sporadic MSS colorectal cancers, resp.), membranous *β*-catenin was associated with a higher number of hypermethylated loci (3.4, 3.5, and 3.8), compared with tumors showing nuclear *β*-catenin (3.1, 2.7, and 2.9). As evident, the association of hypermethylation with *β*-catenin status was stronger among sporadic (MSI plus MSS combined) than Lynch-associated colorectal cancers, and reached statistical significance among the former tumors (3.6 versus 2.8 in sporadic tumors with membranous versus nuclear *β*-catenin, *P* = 0.020 by ANOVA).

## 4. Discussion

Colorectal tumors are dominated by* APC* mutations which almost always cause deletion of both central domains that interact with *β*-catenin and C-terminal domains that interact with the cytoskeleton and play a role in microtubule binding, cell polarity, and chromosome segregation. Hence, deletion of* APC* C-terminal domains is expected to contribute to the chromosomal instability observed in many colorectal cancers. We have analyzed the chromosomal instability in RKO by M-FISH and CGH which revealed almost stable chromosomal number as evidenced by low aneuploid index and low variability of centromere number in RKO similar to the chromosomally stable MSI lines. This finding is consistent with having wild type APC protein. Interestingly, chromosome structure was not stable in RKO as evidenced by high number of rearranged chromosomes which was not expected for MSI cell line. Previously, we have described MSI cell line with specific tendency to acquire structural rearrangements most of which were balanced translocations [30] but the structural rearrangements observed in RKO were mostly unbalanced as seen by M-FISH and reflected by the high copy number changes observed by CGH. The combination of MSI and structural chromosomal instability pattern, observed in RKO, was reported previously in a poorly metastatic cell line KM12C established in nude mice. Interestingly, some clones of this line acquired high metastatic abilities in vivo and showed polyploid karyotypes combined with MSI [31]. The possibility of development of highly metastatic cells from this rare pattern of combined MSI and structural chromosomal instability might have prognostic application and deserves further analysis in clinical studies.

Consistent with the structural instability in RKO, we observed high frequencies of LOH in primary tumors with membranous *β*-catenin at the majority of the tested loci comparable to the LOH frequency in tumors with nuclear *β*-catenin. The LOH analysis also showed that some specific events are selected for in tumors with membranous *β*-catenin (2p25.1, 6p25.1) consistent with the down regulation of nearby genes (*EPCAM*,* SERPINB1*) observed by expression microarray analysis. The loss of* EPCAM* was reported in Lynch syndrome cancers and germline deletion of this gene was associated with predisposition to colorectal cancer in Lynch syndrome [32–34]. This is relevant to the observation that tumors with inactive Wnt/membranous *β*-catenin were overrepresented among familial colon cancer [6]. Regarding the mechanism of structural chromosomal instability in RKO, we searched the list of differentially expressed genes for DNA repair genes' alteration but we could not find good targets with the exception of* ERCC1* and* RECQL* which were rather upregulated in RKO. Overall, the tendency for structural chromosomal instability in RKO justifies more detailed studies to analyze its extent, mechanism, and consequences in the development of this subset of colorectal cancers.

The first observation from the expression microarray was that cluster analysis based upon global gene expression profiling corroborated our hypothesis that RKO cell line is unique as it formed a distinct cluster separate from all other cell lines. The high number of the differentially expressed genes (611) exceeding the twofold change threshold and the large number among these with a very high (>10) fold change (54 upregulated and 80 downregulated, total 134/611 genes) also supports that the differences in the carcinogenic mechanisms between these clusters are substantial. Considering known and common pathways in colorectal carcinogenesis, a major difference between RKO and other cell lines is the *β*-catenin activation status. We cannot exclude the effect of other possible molecular differences (e.g.,* BRAF* mutation status) on the expression pattern of the cell lines.* BRAF* mutation is known to correlate with MSI and CIMP, but RKO and most of other cell lines were MSI-positive and CIMP-positive, regardless of BRAF mutation status; hence this effect remains to be a confounding factor. We characterized our primary tumors for* BRAF* mutation; the frequencies we obtained were in agreement with published reports on the occurrence of* BRAF* V600E mutation in sporadic and Lynch associated colorectal cancers [35–37].

We then proceeded to verify whether or not selected target genes have a role in well characterized categories of primary colorectal cancers. We started by LOH analysis as it was recently reported that LOH is an alternative mechanism that plays a significant role in the pathogenesis of colorectal cancers with chromosomal and microsatellite instability [38]. Overall, the majority of the tested loci were susceptible to LOH in tumors with nuclear as well as membranous *β*-catenin. Some loci showed high frequency of LOH consistent with common areas of gains and losses reported in colorectal cancers such as 7p15.2, 7q22.1, 8p22, 9p21.1, 12p12.3, 12q14.1, 17p13.1, 20q1.21, and 20q13.32 [8, 39]. Some target genes associated with these loci were previously reported as putative tumor suppressor in colorectal cancer including* MUC12* [40],* MTUS1* [38], and* ARFGAP1/SMAP1* [41], while others were novel targets with putative role in colorectal carcinogenesis that deserves further analysis (*NFE2L3*,* DGKI*,* ADAMTSL1*,* RASSF8*,* DCTN2*,* GLIPR1*, and* ASGR1*). Loci that were not commonly reported as targets of gains, losses, or allelic imbalances in colorectal cancers included 1q23.2, 2q11.2, 5q35.2, 11q22.3, and 11q24.1 and their associated target genes also included known tumor suppressors/immune killers such as* IL18* [42],* OPCML/OBCAM* [43], or novel colon cancer genes (*IFI16*,* RGS4*,* DEDD*,* AFF3*,* SORL1*, and* PDLIM7*).

The immunohistochemistry of selected target genes (*IFI16*,* RGS4*,* MCTP1*,* DGKI*,* OBCAM/OPCML*, and* GLIPR1*) in primary tumors confirmed that they were differentially expressed in clinical specimens.* DGKI* and* GLIPR1* showed significant relationship with the *β*-catenin subcellular localization in a pattern similar to that observed by the cDNA expression microarray analysis of the cell lines.* GLIPR1* (glioma pathogenesis-related protein 1) is a p53 target gene that has p53-dependent and p53-independent proapoptotic activities in tumor cells [44]. It is downregulated in glioma and prostate cancer and it could be a potential therapeutic target for prostate cancer [45, 46]. Our data show that it is also a potential tumor suppressor in colorectal cancer and its expression is downregulated in association with *β*-catenin activity.* DGKI* (diacylglycerol kinase, iota) is a member of type IV diacylglycerol kinase subfamily which regulates the intracellular concentration of diacylglycerol through its phosphorylation. The specific role of this particular enzyme is undetermined and very few publications are available on this gene in general. Interestingly,* DGKI* was listed among genes mutated in melanoma as identified by next generation sequencing and also among hypermethylated gene list identified by whole-genome methylation analysis of glioblastoma [47, 48]. Our data suggest that* DGKI* is a novel potential tumor suppressor in colorectal cancers and its expression is downregulated subsequent to *β*-catenin activation too.

**Figure 4:**
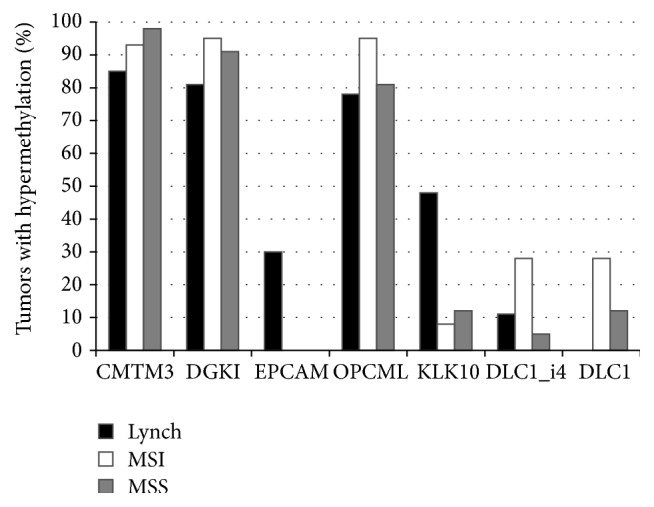
Frequency of promoter hypermethylation for all selected genes in the three categories of tumors. *y*-axis indicates the percentage of tumors showing hypermethylation, based on cut-off values given in Supplementary Table S4.

Then we proceeded with methylation analysis as several lines of evidence suggested close interactions between the epigenetic system and Wnt signaling [49–51]. We selected* CMTM3*,* DGKI*,* DLC1*, and* OPCML* from the list of genes overexpressed in RKO, and* EPCAM* and* KLK10* from the list of genes underexpressed in RKO. As discussed above, LOH and immunohistochemistry supported the role of* DGKI*,* EPCAM*, and* OPCML* in clinical colon cancer specimens. Virtually all the genes were hypermethylated to some degree in clinical specimens of colon cancer, with* CMTM3*,* DGKI*, and* OPCML* showing the highest frequencies (range 78%–98) and the most significant methylation degree compared to the matching normal tissues (Figure 4).* CMTM3* gene belongs to the chemokine-like factor gene superfamily, a novel family that is similar to the chemokine and the transmembrane 4 superfamilies of signaling molecules and it is located at the potential tumor suppressor locus 16q22.1.* CMTM3* was shown to be silenced by promotor methylation in a pilot study of various types of carcinomas including few colon cancer specimens and it was also shown to exert tumor suppressor properties in vitro [52]. This report was corroborated by specific reports on its tumor suppressor properties in clear cell lung cancer [53], gastric cancer [54], and testicular cancer [55].* OPCML/OBCAM* encodes a highly conserved protein localized in the plasma membrane. It is a well-established tumor suppressor of ovarian cancers that is frequently silenced by promoter hypermethylation and the detection of methylation of* OPCML* promoter in ovarian cancer tissues could have prognostic application [56]. It was found to be frequently methylated in other cancers including those of the lung and liver, and it was in the top-ranking hypermethylated genes detected by high density DNA methylation microarray screening of small number of colorectal cancers [57]. The* DGKI* gene was discussed above. Overall, the current evidence of* CMTM3*,* DGKI*, and* OPCML* gene promoter hypermethylation suggests that silencing of these genes is a significant event in colorectal carcinogenesis given the very high frequency of methylated tumors of various categories as well as the significant methylation dosage ratios in tumors compared to normal mucosa.

*KLK10* provided the best example of subgroup-specificity, being hypermethylated in 48% of Lynch syndrome-colorectal cancers compared to 8% for sporadic MSI and 12% for sporadic MSS colorectal cancers. Growing evidence suggests that many kallikreins are implicated in carcinogenesis and some have potential as novel cancer and other disease biomarkers.* KLK10* is one of the fifteen kallikrein subfamily members which encodes a secreted protein and may play a role in suppression of tumorigenesis in breast and prostate cancers. Interestingly,* KLK10* has been recently shown to be associated with prognosis of colon and other cancers [58–60].* EPCAM* was exclusively methylated in Lynch syndrome colorectal cancers with a frequency of 30% which is interesting given that germline deletion of* EPCAM* was reported to predispose to colorectal cancer in Lynch syndrome through methylation of* MSH2* [32–34]. Our data shows that* EPCAM* itself could be also susceptible to methylation in a small subset of Lynch syndrome-related colorectal cancers.

Interestingly, *β*-catenin subcellular localization stratified the tumors into two groups relative to hypermethylation, with membranous (inactive) *β*-catenin being associated with a higher frequency of hypermethylated genes compared to nuclear (active) *β*-catenin. This is an intriguing observation and supports previous evidence implicating epigenetic modifications as one important layer in the multilayered control system responsible for cell-type specific regulation of Wnt target genes [49].

This work analyzed the carcinogenic mechanisms of colorectal cancers that lacks active *β*-catenin/TCF regulated transcription using a model cell line and groups of primary sporadic and hereditary colon cancers. The data illustrate that these cancers might evolve through distinct cytogenetic structural chromosomal instability and also involves distinct groups of cancer-related genes which are susceptible to LOH and methylation. The essential regulatory systems important in colon cancer development (epigenetics, Wnt signaling, and genetic instability) appeared to be closely connected. The differentially expressed genes validated here could be used to develop personalized tumor profiling.

## Supplementary Material

The Supplementary Material consists of 9 components. Supplementary Figure SF1 shows representative examples from the immunohistochemistry of colon cancers. Supplementary Tables S1 to S7 (supplementary Table S3 has 2 parts S3A and S3B) show some detailed methods and results as referred to in the main article and indicated in each table title.

## Figures and Tables

**Table 1:** Cytogenetic characteristics of RKO cell line compared to MSI and MSS cell lines.

Cell line	Aneuploid Index^a^	Variability of centromere number^b^	Rearranged Chromosomes by M-FISH/SKY	Copy number changes by CGH
RKO	2	1	14	9
MSI cell lines	1–8 (3.1)	1–3 (2)	0–5 (2.7)	0–4 (2.4)
MSS cell lines	8–19 (14.2)	6–30 (15.4)	9–24 (15.8)	11–30 (19.4)

^a^The aneuploid index was calculated as the sum of all the consistent chromosomal gains or losses present in the karyotype. Thus, for each monosomy or each trisomy present, a score of 1 was contributed to the aneuploid index. The aneuploid index is different from the modal chromosome number because of cell to cell variability in the presence or absence of a specific chromosome. ^b^The variability of centromere numbers was calculated by counting in each of the examined metaphases the number of copies of each centromere, whether in normal or rearranged copies of a chromosome; noting the percentage of metaphases that have deviations from the modal centromere number; and averaging over all centromeres. The data of the MSS and MSI cell lines, shown as range followed by average, were pooled from the published literature [8, 17, 18] omitting the newest duplicate data in chronological order and excluding the nonclassical cell lines C70, HCA7, and LS411.

**Table 2:** Immunohistochemical evaluation of protein expression of the selected target genes in various tumor categories.

	Gene	Chr	Status in RKO	Pilot study, *n* = 22	% loss in MSS sporadic cancers, *n* = 43 (*P* value^*∗*^)	% loss in MSI sporadic cancers, *n* = 16 (*P* value)	% loss in sporadic adenoma, *n* = 12 (*P* value)	% loss in Lynch-colon cancers, *n* = 38 (*P* value)	% loss in FCCX, *n* = 19 (*P* value)
1	*IFI16*	1q22	Upregulated	Loss 43% not correlated with *β*-catenin localization	ND	ND	ND	ND	ND

2	*RGS4*	1q23.3	Upregulated	RGS4 nuclear localization in 56% of tumors correlated with nuclear *β*-catenin	58% cytoplasmic (NS)	50% cytoplasmic (NS)	ND	ND	ND

3	*MCTP1*	5q15	Upregulated	50% membranous, 50% cytoplasmic, not correlated with *β*-catenin localization	ND	ND	ND	ND	ND

4	*DGKI*	7q32.3–q33	Upregulated	**Loss correlated with ** **β**-**c** **a** **t** **e** **n** **i** **n** **localization**	**55% (0.0001)**	**69% (0.0001)**	**30% (NS)**	**63% (0.0001)**	**50% (0.002)**

5	*OBCAM/OPCML*	11q25	Upregulated	Loss in 55% but not correlated with *β*-catenin localization	60% (NS)	50% (NS)	70% (NS)	68% (NS)	15% (NS)

6	*GLIPR1*	12q21.2	Upregulated	**Loss correlated with ** **β**-**c** **a** **t** **e** **n** **i** **n** **localization**	**33% (0.005)**	**27% (0.005)**	**27% (0.001)**	**44% (0.001)**	**40% (0.035)**

^*∗*^ *P* value is for loss of the expression versus *β*-catenin subcellular localization.

Highlighted in bold are *GLIPR1* and *DGKI* findings which correlated with *β*-catenin status in a pattern similar to that observed in the microarray.

ND, not done; NS, not significant.
