# Supplementary material for: The Role of Chromosomal Instability and Epigenetics in Colorectal Cancers Lacking β-Catenin/TCF Regulated Transcription
Source: Gastroenterol Res Pract. 2016 Mar 7;2016:6089658. doi: 10.1155/2016/6089658 (PMC4800109; doi:10.1155/2016/6089658)
Supplement: Supplementary file 1 — The Supplementary Material consists of 9 components. Supplementary Figure SF1 shows representative examples from the immunohistochemistry of colon cancers. Supplementary Tables S1 to S7 (supplementary Table S3 has 2 parts S3A and S3B) show some detailed methods and results as referred to in the main article and indicated in each table title. [file 6089658.f1.pdf]

Supplementary Figure SF1: Representative examples from the immunohistochemistry of colon cancers. A, membranous β-catenin; B, nuclear β-catenin; C, carcinoma negative for DGKI; D, carcinoma positive for DGKI; E, carcinoma negative for OBCML; F, carcinoma positive for OBCML; E, carcinoma negative for GLIPR1; F, carcinoma negative for GLIPR1. Original magnification x200.

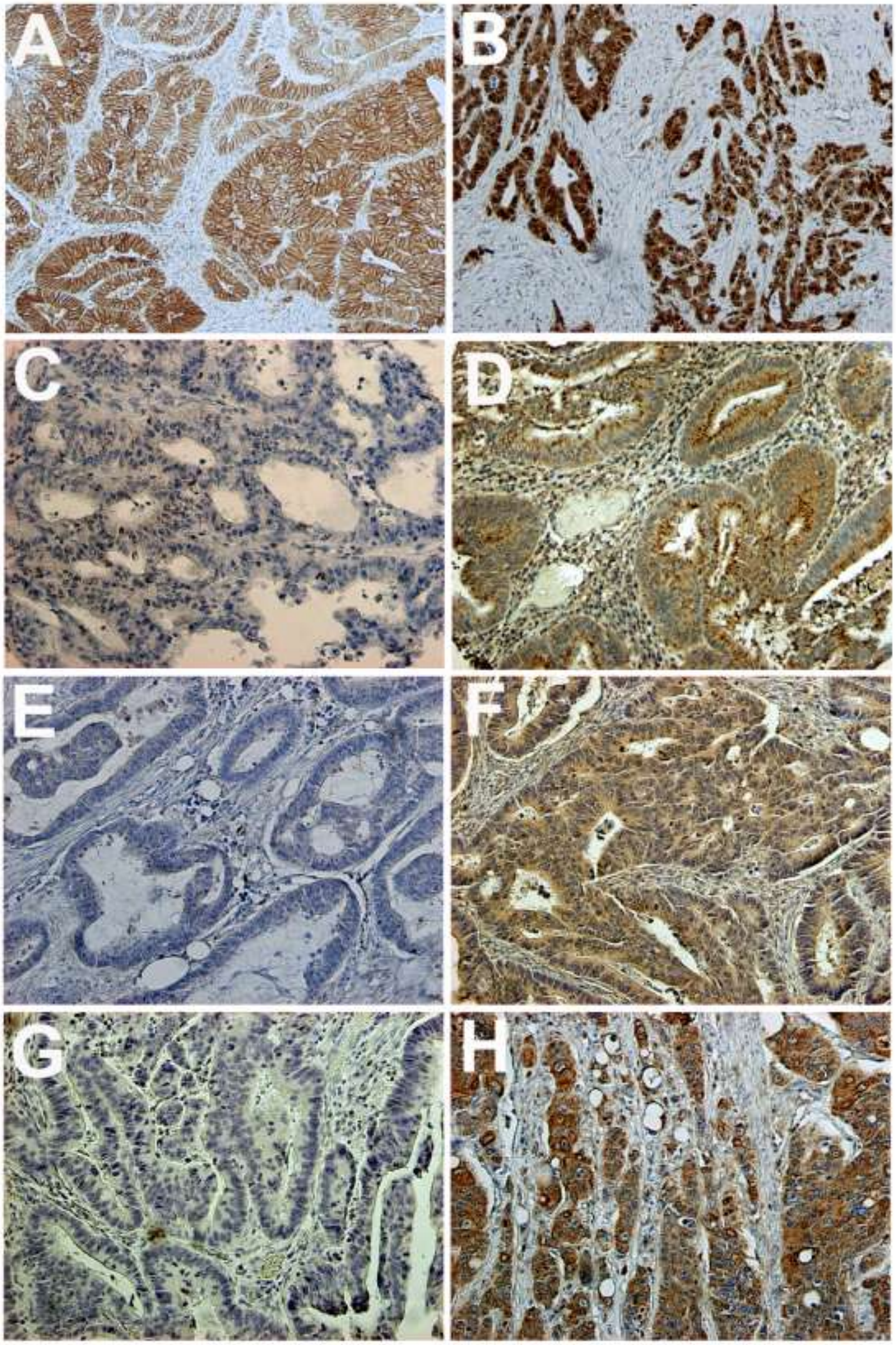

Supplementary table S1. Characteristics of the dinucleotide repeat markers used for LOH analyses and the obtained LOH results

	marker	chr	nearby* differentially expressed gene(s)	LOH % in carcinoma with membranous β-catenin	LOH % in carcinoma with nuclear β-catenin	Overall LOH % in carcinoma
2	D1S2635	1q23.2	IFI16, RGS4, DEDD	44	44	44
1	D1S245	1q32.2	ELF3, GOS2	25	40	33
3	D2S2278	2p25.1	KLF11, LPIN1, EPCAM	43	13	27
4	D2S2264	2q11.2	AFF3	20	60	47
5	D2S2380	2q24.3	IFIH1, GALNT3	0	0	0
6	D2S326	2q31.1	ITGA6	29	40	35
7	D4S3042	4q21.1	EREG, ANXA3	33	38	36
8	D4S426	4q35.2	FAT1, TLR3	25	40	33
9	D5S2111	5q35.2	PDLIM7	20	43	42
10	D6S1640	6p24.3	DSP	17	30	25
11	D6S1617	6p25.1	SERPINB1	43	25	33
12	D7S2564	7p15.2	NFE2L3	75	63	67
13	D7S662	7q22.1	MUC12, DGKI	83	67	73
14	D8S552	8p22	MTUS1, DLC1	38	70	56
15	D8S514	8q24.13	ST3GAL1	40	29	33
16	D9S259	9p21.2	ADAMTSL1	50	50	50
17	D9S288	9p24.2	PDCD1LG2	33	13	21
18	D9S290	9q24.1	NUP188	20	30	27
19	D9S158	9q34.3	ANAPC2, MARPL41, RAPGEF1	29	40	33
20	D11S928	11p15.1	PLEKHA7	0	33	17
21	D11S4159	11q22.3	IL18	33	57	50
22	D11S4167	11q24.1	SORL1, OPCML	20	50	38
23	D12S1688	12p12.1	RECQL	43	25	32
24	D12S1728	12p12.3	RASSF8	40	71	53
25	D12S355	12q14.1	NAB2, DCTN2, GLIPR1	33	56	50
26	D15S983	15q23	CORO2B	29	43	36
27	D17S1353	17p13.1	ASGR1, CLDN1	71	80	76
28	D19S219	19q13.32	ERCC1, KLK10	14	33	22
29	D20S874	20q1.21	ID1	57	73	67
30	D20S171	20q13.32	SLC17A9, ARFGAP1/SMAP1	60	60	60
31	D22S272	22q13.1	RBFOX2	33	33	33

* within 1 megabase on average

Supplementary table S2: The Antibodies used for immunohistochemistry analysis

	Antibody	Host/clonality	Dilution	Antibody supplier
1	IFI16	Mouse monoclonal	1:125	Abnova Corp
2	RGS4	Rabbit polyclonal	1:1000	Atlas-Sigma
3	MCTP1	Rabbit polyclonal	1:200	Atlas-Sigma
4	DGKI	Rabbit polyclonal	1:180	Abgent
5	OBCAM/OPCML	Rabbit polyclonal	1:75	R&D
6	GLIPR1	Rabbit polyclonal	1:100	Atlas-Sigma

Supplementary Table S3A. Sequences for bisulphite sequencing primers

Gene	Orientation	Reference	Sequence (5`- 3`)	Product	Ta[a]
CMTM3_F	forward	This study	TAGGTTAGGAAGTTTTGTAGATGTG	261	52
CMTM3_R	reverse	This study	ATACCCTCTCTTTTTCTTAAAAACC		
DGKI_F	forward	This study	GGTTTTGGTTTTTTTAGTTTTA	372	56
DGKI_R	reverse	This study	ACAAATAACAACCCCTTCCC		
EpCAM_F	forward	This study	GAAGGTTTTTTGTTTGTGTTTGTATT	298	56
EpCAM_R	reverse	This study	AAAAAATAAATAAACTCCCCTCCC		
OPCML_F	forward	(1)	CCACCCCGCCCCTCTGTAGGGGAAGC	250	56
OPCML_R	reverse	(1)	GTGGCGGTCAGGGATGGAGCTGCTG		
KLK10_F	forward	This study	GGTGTTTTGGTGGATTAGAGTTG	290	52
KLK10_R	reverse	This study	TCCTCCTAAAACTCAAAAAAAATCA		
DLC1_i4_F	forward[b]	This study	TTAGGTGAGTTATTGGGTTGTA	409	52
DLC1_i4_BGS1	forward[b]	(2)	GAAAGTTAAAGATAAGGTTATTTG	564	50
DLC1_i4_BGS2	reverse	(2)	CCAAATAACATCCAAAACTCTAA		
DLC1_F	forward[b]	This study	TAAAACCTACRACCCAAACAAC	171	52
DLC1_BGSb1	forward[b]	(3)	CCAAATAAATACCTTATAACCTTTA	458	54
DLC1_BGSb2	reverse	(3)	GAGGTGYGGTTATGTTTTGGT		

[a] Annealing temperature

[b] Either of the two forward primers was combined with the reverse primer for the gene.

(1) Niskakoski, A. *et al*., unpublished data (2014)

(2) Low, JSW *et al*. (2011) A novel isoform of the 8p22 tumor suppressor gene *DLC1* suppresses tumor growth and is frequently silenced in multiple common tumors. *Oncogene*, **16**, 1923-1935.

(3) Seng, TJ *et al.* (2007) The major 8p22 tumor suppressor *DLC1* is frequently silenced by methylation in both endemic and sporadic nasopharyngeal, esophageal, and cervical carcinomas, and inhibits tumor cell colony formation. *Oncogene*, **26**, 934-944.

Supplementary Table S3B. Sequences for MS-MLPA probes

Gene		MS-MLPA probes (5´-3´)	Product size (bp)	Distance of *Hha1* site to coding ATG (bp)	GC%
CMTM3	LPO	GGGTTCCCTAAGGGTTGGATGTGTGTGTGCCTGGAGCT**GCGC**TTGCA	97	-636	61
	RPO	GGTCTGAGAGTCCTCTGAGAATGGGTTTCTAGATTGGATCTTGCTGGCAC			52
DGKI	LPO	GGGTTCCCTAAGGGTTGGACATAGGACCCAGG**GCGC**GGCCACTCGAGTT	102	-306	70
	RPO	CCCCGGACGCTCTCCTTCCTGATGCTTTCATCTAGATTGGATCTTGCTGGCAC			65
EpCAM	LPO	GGGTTCCCTAAGGGTTGGACATCCAAAGATCCCTAACGCCGCCATGGAGACGAAGCA	114	-398	57
	RPO	CCTGGGGCGGGGCGGAGCGGG**GCGC**GCGGGTTCATCTAGATTGGATCTTGCTGGCAC			93
OPCML	LPO	GGGTTCCCTAAGGGTTGGACATCTTGAGTCCATCTAGCGAAGTGG**GCGC**GGTGCGGGCTCAT	119	-1021	70
	RPO	CCCCGCAGGCATCCCCAGCCCGGTGGGTTCATGGTCTAGATTGGATCTTGCTGGCAC			81
KLK10 (1,2)	LPO	GGGTTCCCTAAGGGTTGGACATCTTCCACAGCTGGAGGTGCGGTCCCCGGTGTCCTTCCA	124	+2171	68
	RPO	GCAGGAGGAGAGACCGGGCTT**GCGC**TGTGGCCGCCTTCATGTCTAGATTGGATCTTGCTGGCAC			74
DLC1_i4	LPO	GGGTTCCCTAAGGGTTGGACATCTTGAGTCCATCTTGAGTCCATCTTGTCGCCG**GCGC**AGGGTAAGTC	139	-216	71
	RPO	TTTCCAGATGTGGCCGCCGCGTTCATGGTTCATGGTTCATGGTTCATGTCTAGATTGGATCTTGCTGGCAC			67
DLC1	LPO	GGGTTCCCTAAGGGTTGGACATCTTGAGTCCATCTTGAGTCCATCTTGCTGGCGGAGC**GCGC**GGGAGACA	143	-323	77
	RPO	GTTCGCTCCGACTGCCCGAGCGTTCATGGTTCATGGTTCATGGTTCATGGTCTAGATTGGATCTTGCTGGCAC			73

LPO, left probe oligonucleotide, RPO, right probe oligonucleotide

HhaI site in **bold**

(1) Huang, W. *et al*. (2007) Downregulation and CpG island hypermethylation of NES1/hK10 gene in the pathogenesis of human gastric cancer. *Cancer Lett*, **1**, 78-85.

(2) Lu, C-Y. *et al*. (2009) Aberrant DNA Methylation Profile and Frequent Methylation of *KLK10* and *OXGR1* Genes in Hepatocellular Carcinoma. *Genes Chromosomes Cancer*, **12**, 1057-1068.

Supplementary Table S4.
Normal tissue-based threshold values for the detection of hypermethylation at gene loci in tumor tissues.

Gene	Normal tissue	Average methylation dosage ratio ($\bar{x}_{Dm}$)	Standard deviation	$\bar{x}_{Dm}$+ 1 standard deviation
CMTM3	colorectal mucosa*	0.12	0.08	0.20
DGKI	colorectal mucosa*	0.13	0.06	0.19
EpCAM	colorectal mucosa*	0.13	0.05	0.18
OPCML	colorectal mucosa*	0.20	0.10	0.30
KLK10	colorectal mucosa*	0.09	0.06	0.15
DLC1	colorectal mucosa*	0.09	0.04	0.13
DLC1_i4	colorectal mucosa*	0.06	0.04	0.10

‡ $\bar{x}_{Dm}$ + 1 SD or technical threshold (Dm = 0.15)[a], whichever was higher

* Corresponding to the sporadic MSI, sporadic MSS, and Lynch groups combined (n = 110)

Supplementary Table S5. *APC, CTNNB1 and CIMP* status in the cell lines (all microsatellite unstable) as per the published literature and our laboratory data.

Cell line	*APC*	*CTNNB1*	*CIMP*[a] *status*
RKO	Not found	Not found	Positive (Ahmed et al., 2013; Joensuu et al., 2008; Mouradov et al., 2014)
HCA7	mutant (Abdel-Rahman et al., 2008)	Not found	Positive (Joensuu et al., 2008; Mouradov et al., 2014)
KM12	mutant (Abdel-Rahman et al., 2008)	Not found	Positive (Joensuu et al., 2008; Mouradov et al., 2014)
HCT15	Mutant (Rowan et al., 2000)	Not found	Positive (Ahmed et al., 2013; Joensuu et al., 2008; Mouradov et al., 2014)
HCT116	Not found	Mutant (Morin et al., 1997; Sparks et al., 1998)	Positive (Ahmed et al., 2013; Joensuu et al., 2008; Mouradov et al., 2014)
LIM1215	Not found	Mutant (Sparks et al., 1998)	Negative (Joensuu et al., 2008; Mouradov et al., 2014)
LoVo	Mutant (Huang et al., 1996; Morin et al., 1997)	Not found	Negative (Ahmed et al., 2013; Joensuu et al., 2008; Mouradov et al., 2014)
SW48	Not found	Mutant (Morin et al., 1997; Sparks et al., 1998)	Positive (Ahmed et al., 2013; Joensuu et al., 2008; Mouradov et al., 2014)

[a]CIMP, CpG island methylator phenotype.

References

Abdel-Rahman, W.M., Kalinina, J., Shoman, S., Eissa, S., Ollikainen, M., Elomaa, O., Eliseenkova, A.V., Butzow, R., Mohammadi, M., Peltomaki, P., 2008. Somatic FGF9 mutations in colorectal and endometrial carcinomas associated with membranous beta-catenin. Hum Mutat 29, 390-397.

Ahmed, D., Eide, P.W., Eilertsen, I.A., Danielsen, S.A., Eknaes, M., Hektoen, M., Lind, G.E., Lothe, R.A., 2013. Epigenetic and genetic features of 24 colon cancer cell lines. Oncogenesis 2, e71.

Huang, J., Papadopoulos, N., McKinley, A.J., Farrington, S.M., Curtis, L.J., Wyllie, A.H., Zheng, S., Willson, J.K.V., Markowitz, S.D., Morin, P., Kinzler, K.W., Vogelstein, B., Dunlop, M.G., 1996. *APC* mutations in colorectal tumors with mismatch repair deficiency. Proc. Natl. Acad. Sci. USA 93, 9049-9054.

Joensuu, E.I., Abdel-Rahman, W.M., Ollikainen, M., Ruosaari, S., Knuutila, S., Peltomaki, P., 2008. Epigenetic signatures of familial cancer are characteristic of tumor type and family category. Cancer Res 68, 4597-4605.

Morin, P.J., Sparks, A.B., Korinek, V., Barker, N., Clevers, H., Vogelstein, B., Kinzler, K.W., 1997. Activation of beta-catenin-Tcf signaling in colon cancer by mutations in beta-catenin or APC. Science 275, 1787-1790.

Mouradov, D., Sloggett, C., Jorissen, R.N., Love, C.G., Li, S., Burgess, A.W., Arango, D., Strausberg, R.L., Buchanan, D., Wormald, S., O'Connor, L., Wilding, J.L., Bicknell, D., Tomlinson, I.P., Bodmer, W.F., Mariadason, J.M., Sieber, O.M., 2014. Colorectal cancer cell lines are representative models of the main molecular subtypes of primary cancer. Cancer Res 74, 3238-3247.

Rowan, A.J., Lamlum, H., Ilyas, M., Wheeler, J., Straub, J., Papadopoulou, A., Bicknell, D., Bodmer, W.F., Tomlinson, I.P.M., 2000. APC mutations in sporadic colorectal tumors: A mutational ''hotspot'' and interdependence of the ''two hits''. Proc. Natl. Acad. Sci. USA 97, 3352-3357.

Sparks, A.B., Morin, P.J., Vogelstein, B., Kinzler, K.W., 1998. Mutational analysis of the APC/beta-catenin/Tcf pathway in colorectal cancer. Cancer Res. 58, 1130-1134.

Supplementary table S6. Differentially expressed genes in RKO vs all other cell lines and normal colon samples

	FC (abs)	Gene Symbol	Gene Title
**Genes upregulated in RKO vs all other cell lines and normal colon samples**			
1	2545164.0	FEZ2	fasciculation and elongation protein zeta 2 (zygin II)
2	436.1	BCL2A1	BCL2-related protein A1
3	151.9	SERPINE1	serpin peptidase inhibitor, clade E (nexin, plasminogen activator inhibitor type 1), member 1
4	143.28	G0S2	G0/G1switch 2
5	118.87	MAP1LC3C	microtubule-associated protein 1 light chain 3 gamma
6	111.83	STC1	stanniocalcin 1
7	88.86	HGF	hepatocyte growth factor (hepapoietin A; scatter factor)
8	82.83	LOC645984	uncharacterized LOC645984
9	73.49	RGS4	regulator of G-protein signaling 4
10	58.22	ZEB1	zinc finger E-box binding homeobox 1
11	46.92	IFI16	interferon, gamma-inducible protein 16
12	44.94	GFPT2	glutamine-fructose-6-phosphate transaminase 2
13	44.94	LOC100130938	uncharacterized LOC100130938
14	43.98	LOC152225	uncharacterized LOC152225
15	42.42	IQCG	IQ motif containing G
16	41.31	ZNF883	zinc finger protein 883
17	39.73	MCTP1	multiple C2 domains, transmembrane 1
18	38.50	CACNA2D1	calcium channel, voltage-dependent, alpha 2/delta subunit 1
19	36.91	LCP1	lymphocyte cytosolic protein 1 (L-plastin)
20	29.76	SPHK1	sphingosine kinase 1
21	26.93	LOC100505562	uncharacterized LOC100505562
22	26.49	ZNF300	zinc finger protein 300
23	26.43	CLEC11A	C-type lectin domain family 11, member A
24	23.01	RGMB	RGM domain family, member B
25	22.15	SPOCD1	SPOC domain containing 1
26	20.26	CMTM3	CKLF-like MARVEL transmembrane domain containing 3
27	19.31	CD274	CD274 molecule
28	18.79	ZNF529	zinc finger protein 529
29	17.33	ZNF438	zinc finger protein 438
30	17.14	ARHGAP22	Rho GTPase activating protein 22
31	16.95	ADAMTSL1	ADAMTS-like 1
32	16.61	SEMA3D	sema domain, immunoglobulin domain (Ig), short basic domain, secreted, (semaphorin) 3D
33	16.48	TMEM156	transmembrane protein 156
34	15.41	LTBP2	latent transforming growth factor beta binding protein 2
35	15.25	MCL1	myeloid cell leukemia sequence 1 (BCL2-related)
36	15.15	C2CD2	C2 calcium-dependent domain containing 2

37	14.33	LAMA1	laminin, alpha 1
38	14.22	PTPN7	protein tyrosine phosphatase, non-receptor type 7
39	13.61	PEA15	phosphoprotein enriched in astrocytes 15
40	12.99	SPRY4	sprouty homolog 4 (Drosophila)
41	12.80	ST6GALNAC4	ST6 (alpha-N-acetyl-neuraminyl-2,3-beta-galactosyl-1,3)-N-acetylgalactosaminide alpha-2,6-sialyltransferase 4
42	12.11	AXL	AXL receptor tyrosine kinase
43	12.02	PPP1R18	protein phosphatase 1, regulatory subunit 18
44	11.74	CORO2B	coronin, actin binding protein, 2B
45	11.71	SCARF1	scavenger receptor class F, member 1
46	11.63	FKBP15	FK506 binding protein 15, 133kDa
47	11.42	SMTN	smoothelin
48	11.38	CBLB	Cbl proto-oncogene, E3 ubiquitin protein ligase B
49	11.20	GAP43	growth associated protein 43
50	10.87	PHC2	polyhomeotic homolog 2 (Drosophila)
51	10.72	ZNF474	zinc finger protein 474
52	10.46	NCS1	neuronal calcium sensor 1
53	10.16	ENTPD7	ectonucleoside triphosphate diphosphohydrolase 7
54	10.10	GLIPR1	GLI pathogenesis-related 1
55	9.51	B4GALT6	UDP-Gal:betaGlcNAc beta 1,4- galactosyltransferase, polypeptide 6
56	9.46	C1orf110	chromosome 1 open reading frame 110
57	8.96	LAIR2	leukocyte-associated immunoglobulin-like receptor 2
58	8.70	SMAGP	small cell adhesion glycoprotein
59	8.62	SHC1	SHC (Src homology 2 domain containing) transforming protein 1
60	8.42	MSANTD3	Myb/SANT-like DNA-binding domain containing 3
61	8.40	ASPHD1	aspartate beta-hydroxylase domain containing 1
62	8.00	PPP1R3B	protein phosphatase 1, regulatory subunit 3B
63	7.97	LOC339290	uncharacterized LOC339290
64	7.86	ZNF643	zinc finger protein 643
65	7.65	DGKI	diacylglycerol kinase, iota
66	7.50	DAPK3	death-associated protein kinase 3
67	7.45	LPIN1	lipin 1
68	7.44	DYRK3	dual-specificity tyrosine-(Y)-phosphorylation regulated kinase 3
69	7.44	ELK3	ELK3, ETS-domain protein (SRF accessory protein 2)
70	7.41	TAGLN	transgelin
71	7.20	MAPKAP1	mitogen-activated protein kinase associated protein 1
72	7.15	LOC100130776	uncharacterized LOC100130776
73	7.13	TTN	titin
74	7.01	XIST	X (inactive)-specific transcript (non-protein coding)
75	6.93	ERCC1	excision repair cross-complementing rodent repair deficiency, complementation group 1 (includes overlapping antisense sequence)
76	6.92	PPP1R15A	protein phosphatase 1, regulatory subunit 15A
77	6.88	SGIP1	SH3-domain GRB2-like (endophilin) interacting protein 1
78	6.85	NAV3	neuron navigator 3
79	6.80	RAB3B	RAB3B, member RAS oncogene family

80	6.79	DMRT2	doublesex and mab-3 related transcription factor 2
81	6.78	UAP1	UDP-N-acteylglucosamine pyrophosphorylase 1
82	6.59	RASSF8	Ras association (RalGDS/AF-6) domain family (N-terminal) member 8
83	6.58	ITGA5	integrin, alpha 5 (fibronectin receptor, alpha polypeptide)
84	6.08	SH2B3	SH2B adaptor protein 3
85	6.06	LOC100506267	uncharacterized LOC100506267
86	5.95	NHLH2	nescient helix loop helix 2
87	5.94	IL11	interleukin 11
88	5.88	CCR10	chemokine (C-C motif) receptor 10
89	5.76	SH3GLB2	SH3-domain GRB2-like endophilin B2
90	5.69	ASGR1	asialoglycoprotein receptor 1
91	5.69	LY96	lymphocyte antigen 96
92	5.60	SRFBP1	serum response factor binding protein 1
93	5.56	TMEM194B	transmembrane protein 194B
94	5.41	LOC100126784	uncharacterized LOC100126784
95	5.39	HEY1	hairy/enhancer-of-split related with YRPW motif 1
96	5.38	PDLIM7	PDZ and LIM domain 7 (enigma)
97	5.35	SH3BP5L	SH3-binding domain protein 5-like
98	5.35	CD68	CD68 molecule
99	5.34	BNC2	basonuclin 2
100	5.31	PKN3	protein kinase N3
101	5.20	ADTRP	androgen-dependent TFPI-regulating protein
102	5.07	PCDHB2	protocadherin beta 2
103	5.07	FLOT1	flotillin 1
104	5.02	PLEKHO1	pleckstrin homology domain containing, family O member 1
105	4.98	TGFB1	transforming growth factor, beta 1
106	4.96	ZNF439	zinc finger protein 439
107	4.88	UTS2	urotensin 2
108	4.86	POLR1E	polymerase (RNA) I polypeptide E, 53kDa
109	4.85	FLJ32255	uncharacterized LOC643977
110	4.83	HTATSF1P2	HIV-1 Tat specific factor 1 pseudogene 2
111	4.77	INF2	inverted formin, FH2 and WH2 domain containing
112	4.71	MAST4	microtubule associated serine/threonine kinase family member 4
113	4.70	NUDC	nuclear distribution C homolog (A. nidulans)
114	4.65	ABL2	v-abl Abelson murine leukemia viral oncogene homolog 2
115	4.65	OPLAH	5-oxoprolinase (ATP-hydrolysing)
116	4.64	FSD1L	fibronectin type III and SPRY domain containing 1-like
117	4.63	SFTA1P	surfactant associated 1, pseudogene
118	4.61	ARL9	ADP-ribosylation factor-like 9
119	4.61	PGF	placental growth factor
120	4.60	ZNF672	zinc finger protein 672
121	4.55	DNLZ	DNL-type zinc finger
122	4.54	FMNL3	formin-like 3
123	4.52	AFF3	AF4/FMR2 family, member 3
124	4.46	ZNF284	Zinc finger protein 284

125	4.45	PCDHB7	protocadherin beta 7
126	4.44	MYO10	myosin X
127	4.44	PSMD2	proteasome (prosome, macropain) 26S subunit, non-ATPase, 2
128	4.44	SERPINB8	serpin peptidase inhibitor, clade B (ovalbumin), member 8
129	4.38	ANGPTL4	angiopoietin-like 4
130	4.38	PLAUR	plasminogen activator, urokinase receptor
131	4.37	ZNF404	zinc finger protein 404
132	4.36	RGNEF	190 kDa guanine nucleotide exchange factor
133	4.36	SEMA7A	semaphorin 7A, GPI membrane anchor (John Milton Hagen blood group)
134	4.33	SPSB1	splA/ryanodine receptor domain and SOCS box containing 1
135	4.32	C1orf52	chromosome 1 open reading frame 52
136	4.26	FAM40B	family with sequence similarity 40, member B
137	4.22	RBM19	RNA binding motif protein 19
138	4.20	PMPCA	peptidase (mitochondrial processing) alpha
139	4.15	MED22	mediator complex subunit 22
140	4.12	GPC6	glypican 6
141	4.11	TEX10	testis expressed 10
142	4.07	ARHGAP31	Rho GTPase activating protein 31
143	4.06	CIZ1	CDKN1A interacting zinc finger protein 1
144	4.00	MYL6B	myosin, light chain 6B, alkali, smooth muscle and non-muscle
145	3.99	CD151	CD151 molecule (Raph blood group)
146	3.99	EXOSC4	exosome component 4
147	3.90	PDCD1LG2	programmed cell death 1 ligand 2
148	3.87	VAC14	Vac14 homolog (S. cerevisiae)
149	3.86	LOC646329	uncharacterized LOC646329
150	3.81	LINC00460	Long intergenic non-protein coding RNA 460
151	3.80	COMMD5	COMM domain containing 5
152	3.80	BEST3	bestrophin 3
153	3.76	SMAP2	small ArfGAP2
154	3.76	GNPDA1	glucosamine-6-phosphate deaminase 1
155	3.76	AOX1	aldehyde oxidase 1
156	3.72	BOLA3-AS1	BOLA3 antisense RNA 1 (non-protein coding)
157	3.72	YIF1B	Yip1 interacting factor homolog B (S. cerevisiae)
158	3.70	LOC100287177	uncharacterized LOC100287177
159	3.70	SLC27A4	solute carrier family 27 (fatty acid transporter), member 4
160	3.67	TBC1D13	TBC1 domain family, member 13
161	3.64	RECQL	RecQ protein-like (DNA helicase Q1-like)
162	3.63	DBNDD2///SYS1///SYS1-DBNDD2	dysbindin (dystrobrevin binding protein 1) domain containing 2///SYS1 Golgi-localized integral membrane protein homolog (S. cerevisiae)///SYS1-DBNDD2 readthrough (non-protein coding)
163	3.63	RAPGEF1	Rap guanine nucleotide exchange factor (GEF) 1
164	3.61	SLC9B2	solute carrier family 9, subfamily B (NHA2, cation proton antiporter 2), member 2
165	3.59	OPCML	opioid binding protein/cell adhesion molecule-like
166	3.55	C1orf51	chromosome 1 open reading frame 51
167	3.53	BAK1	BCL2-antagonist/killer 1

168	3.52	EFTUD1	elongation factor Tu GTP binding domain containing 1
169	3.51	CCDC85B	coiled-coil domain containing 85B
170	3.50	RIPK2	receptor-interacting serine-threonine kinase 2
171	3.48	TPP1	tripeptidyl peptidase I
172	3.48	ANKS6	ankyrin repeat and sterile alpha motif domain containing 6
173	3.47	CDC42SE1	CDC42 small effector 1
174	3.44	C20orf27	chromosome 20 open reading frame 27
175	3.43	TOP1MT	topoisomerase (DNA) I, mitochondrial
176	3.42	SPATA2	spermatogenesis associated 2
177	3.41	TRAPPC10	trafficking protein particle complex 10
178	3.40	COQ4	coenzyme Q4 homolog (S. cerevisiae)
179	3.39	CDK17	cyclin-dependent kinase 17
180	3.38	FAM176B	family with sequence similarity 176, member B
181	3.38	WDFY2	WD repeat and FYVE domain containing 2
182	3.37	FRMD4A	FERM domain containing 4A
183	3.37	OGFR	opioid growth factor receptor
184	3.32	ASB6	ankyrin repeat and SOCS box containing 6
185	3.32	RBFOX2	RNA binding protein, fox-1 homolog (C. elegans) 2
186	3.30	CAMK2N2	calcium/calmodulin-dependent protein kinase II inhibitor 2
187	3.29	TOMM34	translocase of outer mitochondrial membrane 34
188	3.28	SLC9A1	solute carrier family 9, subfamily A (NHE1, cation proton antiporter 1), member 1
189	3.27	HGS	hepatocyte growth factor-regulated tyrosine kinase substrate
190	3.27	NAB2	NGFI-A binding protein 2 (EGR1 binding protein 2)
191	3.24	NTMT1	N-terminal Xaa-Pro-Lys N-methyltransferase 1
192	3.24	TP53RK	TP53 regulating kinase
193	3.22	GSTO1	glutathione S-transferase omega 1
194	3.21	PQLC2	PQ loop repeat containing 2
195	3.20	ARF3	ADP-ribosylation factor 3
196	3.19	HMGA2	high mobility group AT-hook 2
197	3.18	DUSP3	dual specificity phosphatase 3
198	3.18	COBRA1	cofactor of BRCA1
199	3.16	LOC401588	uncharacterized LOC401588
200	3.16	UCK1	uridine-cytidine kinase 1
201	3.15	FPGS	folylpolyglutamate synthase
202	3.15	B3GAT1	beta-1,3-glucuronyltransferase 1 (glucuronosyltransferase P)
203	3.14	FGFR1	fibroblast growth factor receptor 1
204	3.13	DLGAP4	discs, large (Drosophila) homolog-associated protein 4
205	3.13	BCL10	B-cell CLL/lymphoma 10
206	3.13	CENPT	centromere protein T
207	3.12	PUF60	poly-U binding splicing factor 60KDa
208	3.11	CPQ	carboxypeptidase Q
209	3.09	GNB2	guanine nucleotide binding protein (G protein), beta polypeptide 2
210	3.09	VEGFB	vascular endothelial growth factor B
211	3.07	PPM1J	protein phosphatase, Mg2+/Mn2+ dependent, 1J

212	3.06	ABCF2	ATP-binding cassette, sub-family F (GCN20), member 2
213	3.06	MFSD10	major facilitator superfamily domain containing 10
214	3.05	LBX2-AS1	LBX2 antisense RNA 1 (non-protein coding)
215	3.05	VPS53	vacuolar protein sorting 53 homolog (S. cerevisiae)
216	3.05	STK4	serine/threonine kinase 4
217	3.05	HOMER3	homer homolog 3 (Drosophila)
218	3.04	PNP	purine nucleoside phosphorylase
219	3.04	RAD51L3-RFFL///RFFL	RAD51L3-RFFL readthrough///ring finger and FYVE-like domain containing E3 ubiquitin protein ligase
220	3.00	TTLL11	tubulin tyrosine ligase-like family, member 11
221	2.99	TOR1A	torsin family 1, member A (torsin A)
222	2.99	MICAL1	microtubule associated monoxygenase, calponin and LIM domain containing 1
223	2.99	ATAD3A///ATAD3B	ATPase family, AAA domain containing 3A///ATPase family, AAA domain containing 3B
224	2.99	M6PR	mannose-6-phosphate receptor (cation dependent)
225	2.98	ZC3H3	zinc finger CCCH-type containing 3
226	2.97	STX1A	syntaxin 1A (brain)
227	2.97	CD320	CD320 molecule
228	2.96	NSMAF	neutral sphingomyelinase (N-SMase) activation associated factor
229	2.96	MAZ	MYC-associated zinc finger protein (purine-binding transcription factor)
230	2.95	MED27	mediator complex subunit 27
231	2.95	ANTXR2	anthrax toxin receptor 2
232	2.92	DGKG	diacylglycerol kinase, gamma 90kDa
233	2.88	PPA2	pyrophosphatase (inorganic) 2
234	2.88	CTTNBP2NL	CTTNBP2 N-terminal like
235	2.87	CXorf40A///CXorf40B	chromosome X open reading frame 40A///chromosome X open reading frame 40B
236	2.87	C8orf31	chromosome 8 open reading frame 31
237	2.85	SF3B3	splicing factor 3b, subunit 3, 130kDa
238	2.85	DCTN5	dynactin 5 (p25)
239	2.84	NUP188	nucleoporin 188kDa
240	2.83	ANAPC2	anaphase promoting complex subunit 2
241	2.82	MUC12	mucin 12, cell surface associated
242	2.82	RNF208	ring finger protein 208
243	2.82	HERC2P3	hect domain and RLD 2 pseudogene 3
244	2.81	LRWD1///MIR4467	leucine-rich repeats and WD repeat domain containing 1///microRNA 4467
245	2.80	LCAT	lecithin-cholesterol acyltransferase
246	2.80	MYO5A	myosin VA (heavy chain 12, myoxin)
247	2.80	PEX5	peroxisomal biogenesis factor 5
248	2.79	CDC37	cell division cycle 37 homolog (S. cerevisiae)
249	2.78	COPS7A	COP9 constitutive photomorphogenic homolog subunit 7A (Arabidopsis)
250	2.78	NXPE3	neurexophilin and PC-esterase domain family, member 3
251	2.77	SSSCA1	Sjogren syndrome/scleroderma autoantigen 1

252	2.76	PIGT	phosphatidylinositol glycan anchor biosynthesis, class T
253	2.75	C17orf81	chromosome 17 open reading frame 81
254	2.75	HSD17B14	hydroxysteroid (17-beta) dehydrogenase 14
255	2.75	CCND3	cyclin D3
256	2.75	PCOLCE	procollagen C-endopeptidase enhancer
257	2.73	SSNA1	Sjogren syndrome nuclear autoantigen 1
258	2.72	COPS6	COP9 constitutive photomorphogenic homolog subunit 6 (Arabidopsis)
259	2.72	SAT2	spermidine/spermine N1-acetyltransferase family member 2
260	2.71	ZNF167	zinc finger protein 167
261	2.70	USP20	ubiquitin specific peptidase 20
262	2.70	NLRP11	NLR family, pyrin domain containing 11
263	2.69	TEAD2	TEA domain family member 2
264	2.68	VPS4A	vacuolar protein sorting 4 homolog A (S. cerevisiae)
265	2.67	MOCS3	molybdenum cofactor synthesis 3
266	2.67	SURF4	surfeit 4
267	2.66	C19orf43	chromosome 19 open reading frame 43
268	2.65	IKBKB	inhibitor of kappa light polypeptide gene enhancer in B-cells, kinase beta
269	2.65	RNF24	ring finger protein 24
270	2.63	LONRF3	LON peptidase N-terminal domain and ring finger 3
271	2.63	MEPCE	methylphosphate capping enzyme
272	2.62	FASTKD5	FAST kinase domains 5
273	2.62	NIPA1	non imprinted in Prader-Willi/Angelman syndrome 1
274	2.61	RIC8A	resistance to inhibitors of cholinesterase 8 homolog A (C. elegans)
275	2.61	HSF1	heat shock transcription factor 1
276	2.61	PDE4D	phosphodiesterase 4D, cAMP-specific
277	2.61	SLC9A7	solute carrier family 9, subfamily A (NHE7, cation proton antiporter 7), member 7
278	2.60	DDX31	DEAD (Asp-Glu-Ala-Asp) box polypeptide 31
279	2.60	FBXW2	F-box and WD repeat domain containing 2
280	2.59	CTU2	cytosolic thiouridylase subunit 2 homolog (S. pombe)
281	2.59	VEGFC	vascular endothelial growth factor C
282	2.59	C9orf78	chromosome 9 open reading frame 78
283	2.58	RAB3IP	RAB3A interacting protein (rabin3)
284	2.56	LOC728485	uncharacterized LOC728485
285	2.56	POLR1A	polymerase (RNA) I polypeptide A, 194kDa
286	2.53	C20orf112	chromosome 20 open reading frame 112
287	2.53	KCNH2	potassium voltage-gated channel, subfamily H (eag-related), member 2
288	2.53	TMEM203	transmembrane protein 203
289	2.53	BAZ2A	bromodomain adjacent to zinc finger domain, 2A
290	2.53	TRAF2	TNF receptor-associated factor 2
291	2.52	GTF3C5	general transcription factor IIIC, polypeptide 5, 63kDa
292	2.52	ASB1	ankyrin repeat and SOCS box containing 1
293	2.51	FAM65A	family with sequence similarity 65, member A
294	2.51	SNRPB	small nuclear ribonucleoprotein polypeptides B and B1
295	2.51	SLC17A9	solute carrier family 17, member 9

296	2.51	SMURF1	SMAD specific E3 ubiquitin protein ligase 1
297	2.50	MRAP2	melanocortin 2 receptor accessory protein 2
298	2.50	PSMB7	proteasome (prosome, macropain) subunit, beta type, 7
299	2.49	EVC	Ellis van Creveld syndrome
300	2.49	CERS5	ceramide synthase 5
301	2.49	ZNF252P	zinc finger protein 252, pseudogene
302	2.49	SLC39A13	solute carrier family 39 (zinc transporter), member 13
303	2.48	CCDC136	coiled-coil domain containing 136
304	2.48	MYO9B	myosin IXB
305	2.48	RUSC2	RUN and SH3 domain containing 2
306	2.48	PBX2	pre-B-cell leukemia homeobox 2
307	2.47	ETV4	ets variant 4
308	2.47	ZNF583	zinc finger protein 583
309	2.47	EXOG	endo/exonuclease (5'-3'), endonuclease G-like
310	2.46	MAPKAPK3	mitogen-activated protein kinase-activated protein kinase 3
311	2.46	PI4K2A	phosphatidylinositol 4-kinase type 2 alpha
312	2.45	RALGDS	ral guanine nucleotide dissociation stimulator
313	2.44	ADRM1	adhesion regulating molecule 1
314	2.43	GIT2	G protein-coupled receptor kinase interacting ArfGAP 2
315	2.43	ZMAT5	zinc finger, matrin-type 5
316	2.43	HM13	histocompatibility (minor) 13
317	2.43	TRPC4AP	transient receptor potential cation channel, subfamily C, member 4 associated protein
318	2.40	PCDHB8	protocadherin beta 8
319	2.40	SMG9	smg-9 homolog, nonsense mediated mRNA decay factor (C. elegans)
320	2.40	TLR6	toll-like receptor 6
321	2.40	TOLLIP	toll interacting protein
322	2.39	TMEM145	transmembrane protein 145
323	2.38	ELK1	ELK1, member of ETS oncogene family
324	2.38	ZNF71	zinc finger protein 71
325	2.38	CEP250	centrosomal protein 250kDa
326	2.36	FOXK2	forkhead box K2
327	2.36	ZNF597	zinc finger protein 597
328	2.36	MAP1LC3B	microtubule-associated protein 1 light chain 3 beta
329	2.35	TLE3	transducin-like enhancer of split 3 (E(sp1) homolog, Drosophila)
330	2.35	ATXN7L2	ataxin 7-like 2
331	2.34	FLJ26850	FLJ26850 protein
332	2.34	SLC15A3	solute carrier family 15, member 3
333	2.34	TMEM138	transmembrane protein 138
334	2.34	SRRT	serrate RNA effector molecule homolog (Arabidopsis)
335	2.32	TMEM246	transmembrane protein 246
336	2.31	BCL7C	B-cell CLL/lymphoma 7C
337	2.28	APH1B	anterior pharynx defective 1 homolog B (C. elegans)
338	2.28	ARPC5L	actin related protein 2/3 complex, subunit 5-like
339	2.28	HES7	hairy and enhancer of split 7 (Drosophila)

340	2.27	TOR1B	torsin family 1, member B (torsin B)
341	2.27	UFC1	ubiquitin-fold modifier conjugating enzyme 1
342	2.26	LEPRE1	leucine proline-enriched proteoglycan (leprecan) 1
343	2.25	DUS2L	dihydrouridine synthase 2-like, SMM1 homolog (S. cerevisiae)
344	2.25	SNORA5B///TBRG4	small nucleolar RNA, H/ACA box 5B///transforming growth factor beta regulator 4
345	2.25	EIF5A	eukaryotic translation initiation factor 5A
346	2.23	C14orf135	chromosome 14 open reading frame 135
347	2.22	TFR2	transferrin receptor 2
348	2.22	ARMC9	armadillo repeat containing 9
349	2.21	WDR85	WD repeat domain 85
350	2.21	RNF220	ring finger protein 220
351	2.20	DDAH1	dimethylarginine dimethylaminohydrolase 1
352	2.20	DAXX	death-domain associated protein
353	2.20	PCDHB16	protocadherin beta 16
354	2.20	SEC61A2	Sec61 alpha 2 subunit (S. cerevisiae)
355	2.19	SH2B2	SH2B adaptor protein 2
356	2.19	IRF5	interferon regulatory factor 5
357	2.18	MRPL41	mitochondrial ribosomal protein L41
358	2.17	LOC282997	uncharacterized LOC282997
359	2.17	TBC1D10A	TBC1 domain family, member 10A
360	2.17	SMYD5	SMYD family member 5
361	2.17	SURF2	surfeit 2
362	2.16	ZFYVE1	zinc finger, FYVE domain containing 1
363	2.15	ARFGAP1	ADP-ribosylation factor GTPase activating protein 1
364	2.15	ZNF668	zinc finger protein 668
365	2.15	FXR2	fragile X mental retardation, autosomal homolog 2
366	2.14	DCTN2	dynactin 2 (p50)
367	2.14	ST3GAL1	ST3 beta-galactoside alpha-2,3-sialyltransferase 1
368	2.13	DOK1	docking protein 1, 62kDa (downstream of tyrosine kinase 1)
369	2.13	ZER1	zer-1 homolog (C. elegans)
370	2.13	DEDD	death effector domain containing
371	2.13	SLC35C2	solute carrier family 35, member C2
372	2.13	DRG2	developmentally regulated GTP binding protein 2
373	2.13	CREM	cAMP responsive element modulator
374	2.11	LTK	leukocyte receptor tyrosine kinase
375	2.10	SH3GL1	SH3-domain GRB2-like 1
376	2.09	SLC4A8	solute carrier family 4, sodium bicarbonate cotransporter, member 8
377	2.08	POLR2E	polymerase (RNA) II (DNA directed) polypeptide E, 25kDa
378	2.08	FAM195B	family with sequence similarity 195, member B
379	2.07	PCDHB12	protocadherin beta 12
380	2.07	MRPL15	mitochondrial ribosomal protein L15
381	2.06	FHL3	four and a half LIM domains 3
382	2.06	BAP1	BRCA1 associated protein-1 (ubiquitin carboxy-terminal hydrolase)
383	2.06	PI4KB	phosphatidylinositol 4-kinase, catalytic, beta

384	2.05	LOC344595	uncharacterized LOC344595
385	2.05	SHANK1	SH3 and multiple ankyrin repeat domains 1
386	2.05	SSH2	slingshot homolog 2 (Drosophila)
387	2.05	UBTF	upstream binding transcription factor, RNA polymerase I
388	2.04	MED12	mediator complex subunit 12
389	2.04	PSMD8	proteasome (prosome, macropain) 26S subunit, non-ATPase, 8
390	2.04	SNX21	sorting nexin family member 21
391	2.03	TAF6	TAF6 RNA polymerase II, TATA box binding protein (TBP)-associated factor, 80kDa
392	2.02	CPSF3L	cleavage and polyadenylation specific factor 3-like
393	2.02	ABCD1	ATP-binding cassette, sub-family D (ALD), member 1
394	2.02	DHX38	DEAH (Asp-Glu-Ala-His) box polypeptide 38
395	2.02	FIBP	fibroblast growth factor (acidic) intracellular binding protein
396	2.01	KLHL29	kelch-like 29 (Drosophila)
397	2.00	NCBP1	nuclear cap binding protein subunit 1, 80kDa
398	2.00	SEC22C	SEC22 vesicle trafficking protein homolog C (S. cerevisiae)
399	2.00	FGD3	FYVE, RhoGEF and PH domain containing 3

Genes downregulated in RKO vs all other cell lines and normal colon samples

1	331.08	ANXA3	annexin A3
2	260.31	KRT19	keratin 19
3	255.30	CDH1	cadherin 1, type 1, E-cadherin (epithelial)
4	251.15	MAL2	mal, T-cell differentiation protein 2 (gene/pseudogene)
5	225.92	EREG	epiregulin
6	217.99	AREG///AREGB	amphiregulin///amphiregulin B
7	121.49	FOXQ1	forkhead box Q1
8	98.90	KLF5	Kruppel-like factor 5 (intestinal)
9	95.66	ME1	malic enzyme 1, NADP(+)-dependent, cytosolic
10	89.49	ESRP1	epithelial splicing regulatory protein 1
11	89.39	PLK2	polo-like kinase 2
12	87.90	GALNT3	UDP-N-acetyl-alpha-D-galactosamine:polypeptide N-acetylgalactosaminyltransferase 3 (GalNAc-T3)
13	86.48	CXADR	coxsackie virus and adenovirus receptor
14	75.01	ID2	inhibitor of DNA binding 2, dominant negative helix-loop-helix protein
15	70.91	ATP8B1	ATPase, aminophospholipid transporter, class I, type 8B, member 1
16	70.40	GJB2	gap junction protein, beta 2, 26kDa
17	69.12	PDLIM1	PDZ and LIM domain 1
18	66.28	TSPAN13	tetraspanin 13
19	60.65	SOX9	SRY (sex determining region Y)-box 9
20	60.44	RBM47	RNA binding motif protein 47
21	56.54	KITLG	KIT ligand
22	54.76	RAB25	RAB25, member RAS oncogene family
23	50.73	SCOC	short coiled-coil protein
24	50.00	IL18	interleukin 18 (interferon-gamma-inducing factor)
25	48.74	SNX18	sorting nexin 18

26	47.12	S100A14	S100 calcium binding protein A14
27	44.88	ARL4C	ADP-ribosylation factor-like 4C
28	44.07	F2RL1	coagulation factor II (thrombin) receptor-like 1
29	39.54	SCRN1	secernin 1
30	38.97	TNS3	tensin 3
31	34.37	FAM135A	family with sequence similarity 135, member A
32	33.27	CDS1	CDP-diacylglycerol synthase (phosphatidate cytidylyltransferase) 1
33	32.53	AIM1	absent in melanoma 1
34	31.93	TRIM59	tripartite motif containing 59
35	30.38	TJP2	tight junction protein 2 (zona occludens 2)
36	29.32	ID1	inhibitor of DNA binding 1, dominant negative helix-loop-helix protein
37	29.26	KLK10	kallikrein-related peptidase 10
38	28.80	HES1	hairy and enhancer of split 1, (Drosophila)
39	28.38	SORL1	sortilin-related receptor, L(DLR class) A repeats containing
40	28.37	SHROOM3	shroom family member 3
41	28.29	GPRC5A	G protein-coupled receptor, family C, group 5, member A
42	26.07	KRT8	keratin 8
43	25.36	AHR	aryl hydrocarbon receptor
44	24.62	DSC2	desmocollin 2
45	24.06	MYOF	myoferlin
46	23.35	NUP62CL	nucleoporin 62kDa C-terminal like
47	23.34	ZBTB10	zinc finger and BTB domain containing 10
48	22.08	LGALS3BP	lectin, galactoside-binding, soluble, 3 binding protein
49	21.37	NEBL	nebulette
50	20.76	MYO5B	myosin VB
51	19.61	CLDN7	claudin 7
52	19.59	EPCAM	epithelial cell adhesion molecule
53	19.42	ITGA6	integrin, alpha 6
54	18.52	MYO1D	myosin ID
55	18.39	LOC100499467	uncharacterized LOC100499467
56	17.89	ATXN1	ataxin 1
57	17.65	MPZL2	myelin protein zero-like 2
58	17.62	ADD3	adducin 3 (gamma)
59	17.48	SPINT2	serine peptidase inhibitor, Kunitz type, 2
60	17.25	GRHL2	grainyhead-like 2 (Drosophila)
61	16.14	PIR	pirin (iron-binding nuclear protein)
62	16.07	ARHGAP8///PRR5-ARHGAP8	Rho GTPase activating protein 8///PRR5-ARHGAP8 readthrough
63	15.48	INADL	InaD-like (Drosophila)
64	14.35	EHF	ets homologous factor
65	14.07	ABCC3	ATP-binding cassette, sub-family C (CFTR/MRP), member 3
66	14.07	ELF3	E74-like factor 3 (ets domain transcription factor, epithelial-specific)
67	13.97	JUP	junction plakoglobin
68	13.86	HMMR	hyaluronan-mediated motility receptor (RHAMM)
69	12.92	SERINC5	serine incorporator 5

70	12.84	ENPP5	ectonucleotide pyrophosphatase/phosphodiesterase 5 (putative)
71	12.66	EPB41L5	erythrocyte membrane protein band 4.1 like 5
72	12.43	BTC	betacellulin
73	12.31	CDH3	cadherin 3, type 1, P-cadherin (placental)
74	12.21	C1orf106	chromosome 1 open reading frame 106
75	11.97	DSP	desmoplakin
76	10.90	RNF43	ring finger protein 43
77	10.81	IFIH1	interferon induced with helicase C domain 1
78	10.47	NR1D2	nuclear receptor subfamily 1, group D, member 2
79	10.21	MTUS1	microtubule associated tumor suppressor 1
80	10.04	PPL	periplakin
81	9.99	FAT1	FAT tumor suppressor homolog 1 (Drosophila)
82	9.73	ENDOD1	endonuclease domain containing 1
83	9.71	LAMC2	laminin, gamma 2
84	9.60	CASK	calcium/calmodulin-dependent serine protein kinase (MAGUK family)
85	9.42	AP1M2	adaptor-related protein complex 1, mu 2 subunit
86	9.38	KRTCAP3	keratinocyte associated protein 3
87	9.11	IRF6	interferon regulatory factor 6
88	9.09	GPR39	G protein-coupled receptor 39
89	8.81	C4orf34	chromosome 4 open reading frame 34
90	8.63	SEC63	SEC63 homolog (S. cerevisiae)
91	8.28	SERPINB1	serpin peptidase inhibitor, clade B (ovalbumin), member 1
92	8.17	NFE2L3	nuclear factor (erythroid-derived 2)-like 3
93	8.08	SH2D3A	SH2 domain containing 3A
94	7.94	ATP2B1	ATPase, Ca++ transporting, plasma membrane 1
95	7.72	DENND2D	DENN/MADD domain containing 2D
96	7.66	MYO6	myosin VI
97	7.64	MACC1	metastasis associated in colon cancer 1
98	7.35	FAM59A	family with sequence similarity 59, member A
99	7.29	STXBP6	syntaxin binding protein 6 (amisyn)
100	7.20	SGK223	homolog of rat pragma of Rnd2
101	7.16	NXN	nucleoredoxin
102	6.93	HIBCH	3-hydroxyisobutyryl-CoA hydrolase
103	6.92	DCP1A	DCP1 decapping enzyme homolog A (S. cerevisiae)
104	6.91	GCA	grancalcin, EF-hand calcium binding protein
105	6.85	SH3YL1	SH3 domain containing, Ysc84-like 1 (S. cerevisiae)
106	6.60	UACA	uveal autoantigen with coiled-coil domains and ankyrin repeats
107	6.56	SNTB1	syntrophin, beta 1 (dystrophin-associated protein A1, 59kDa, basic component 1)
108	6.48	SRD5A1	steroid-5-alpha-reductase, alpha polypeptide 1 (3-oxo-5 alpha-steroid delta 4-dehydrogenase alpha 1)
109	6.48	TBC1D8	TBC1 domain family, member 8 (with GRAM domain)
110	6.21	CLOCK	clock homolog (mouse)
111	6.18	TPD52L1	tumor protein D52-like 1
112	6.17	PLEKHA7	pleckstrin homology domain containing, family A member 7

113	6.00	PPP3CA	protein phosphatase 3, catalytic subunit, alpha isozyme
114	5.97	TMC4	transmembrane channel-like 4
115	5.93	ST14	suppression of tumorigenicity 14 (colon carcinoma)
116	5.92	BSPRY	B-box and SPRY domain containing
117	5.82	MFSD6	major facilitator superfamily domain containing 6
118	5.79	USP53	ubiquitin specific peptidase 53
119	5.77	MPZL3	myelin protein zero-like 3
120	5.70	SLC44A5	solute carrier family 44, member 5
121	5.69	OSBPL10	oxysterol binding protein-like 10
122	5.56	C19orf33	chromosome 19 open reading frame 33
123	5.51	BOLA1	bolA homolog 1 (E. coli)
124	5.45	KRT18	keratin 18
125	5.45	TLR3	toll-like receptor 3
126	5.35	JPH1	junctophilin 1
127	5.23	THAP9-AS1	THAP9 antisense RNA 1 (non-protein coding)
128	5.10	KLF11	Kruppel-like factor 11
129	5.09	ELL3	elongation factor RNA polymerase II-like 3
130	5.08	THAP10	THAP domain containing 10
131	5.03	TNFAIP8	tumor necrosis factor, alpha-induced protein 8
132	4.96	LRP11	low density lipoprotein receptor-related protein 11
133	4.82	SFN	stratifin
134	4.82	RIPK4	receptor-interacting serine-threonine kinase 4
135	4.80	MAP7	microtubule-associated protein 7
136	4.79	RHPN2	rhophilin, Rho GTPase binding protein 2
137	4.73	MARVELD2	MARVEL domain containing 2
138	4.63	OSBPL3	oxysterol binding protein-like 3
139	4.58	FA2H	fatty acid 2-hydroxylase
140	4.58	CADPS2	Ca++-dependent secretion activator 2
141	4.56	CBLC	Cbl proto-oncogene, E3 ubiquitin protein ligase C
142	4.42	ISL2	ISL LIM homeobox 2
143	4.40	C16orf95	chromosome 16 open reading frame 95
144	4.35	KIAA0240	KIAA0240
145	4.29	NADKD1	NAD kinase domain containing 1
146	4.19	MYO5C	myosin VC
147	4.15	MAN2A1	mannosidase, alpha, class 2A, member 1
148	4.15	C18orf32///RPL17-C18ORF32	chromosome 18 open reading frame 32///RPL17-C18orf32 readthrough
149	4.14	F11R	F11 receptor
150	4.12	PPP3CC	protein phosphatase 3, catalytic subunit, gamma isozyme
151	4.10	IGIP	IgA-inducing protein homolog (Bos taurus)
152	4.04	ESYT2	extended synaptotagmin-like protein 2
153	3.97	MTCH1	mitochondrial carrier 1
154	3.91	DOCK9	dedicator of cytokinesis 9
155	3.91	FKBP9	FK506 binding protein 9, 63 kDa
156	3.80	FTH1	ferritin, heavy polypeptide 1

157	3.78	PLA2G12A	phospholipase A2, group XIIA
158	3.75	RABGAP1L	RAB GTPase activating protein 1-like
159	3.74	MTR	5-methyltetrahydrofolate-homocysteine methyltransferase
160	3.67	S100A10	S100 calcium binding protein A10
161	3.53	TES	testis derived transcript (3 LIM domains)
162	3.47	FAM177A1	family with sequence similarity 177, member A1
163	3.45	PERP	PERP, TP53 apoptosis effector
164	3.44	PLEKHA1	pleckstrin homology domain containing, family A (phosphoinositide binding specific) member 1
165	3.43	RAB17	RAB17, member RAS oncogene family
166	3.35	C1orf172	chromosome 1 open reading frame 172
167	3.34	ADI1	acireductone dioxygenase 1
168	3.30	BCL2L11	BCL2-like 11 (apoptosis facilitator)
169	3.29	SUN1	Sad1 and UNC84 domain containing 1
170	3.24	AKTIP	AKT interacting protein
171	3.23	MARVELD3	MARVEL domain containing 3
172	3.23	ABCC10	ATP-binding cassette, sub-family C (CFTR/MRP), member 10
173	3.15	PPP1R21	protein phosphatase 1, regulatory subunit 21
174	3.14	DSG2	desmoglein 2
175	3.13	PHKB	phosphorylase kinase, beta
176	3.11	FTH1P5	ferritin, heavy polypeptide 1 pseudogene 5
177	3.06	MBP	myelin basic protein
178	2.97	GCLC	glutamate-cysteine ligase, catalytic subunit
179	2.96	ALDH3A2	aldehyde dehydrogenase 3 family, member A2
180	2.96	TCEB3	transcription elongation factor B (SIII), polypeptide 3 (110kDa, elongin A)
181	2.95	JRKL	jerky homolog-like (mouse)
182	2.91	GFPT1	glutamine--fructose-6-phosphate transaminase 1
183	2.83	FAM149B1	family with sequence similarity 149, member B1
184	2.78	DDR1///MIR4640	discoidin domain receptor tyrosine kinase 1///microRNA 4640
185	2.75	RALGAPA1	Ral GTPase activating protein, alpha subunit 1 (catalytic)
186	2.75	PLEKHG4	pleckstrin homology domain containing, family G (with RhoGef domain) member 4
187	2.72	CRB3	crumbs homolog 3 (Drosophila)
188	2.72	OTUD1	OTU domain containing 1
189	2.65	P4HTM	prolyl 4-hydroxylase, transmembrane (endoplasmic reticulum)
190	2.45	EPM2A	epilepsy, progressive myoclonus type 2A, Lafora disease (laforin)
191	2.42	BOD1L1	biorientation of chromosomes in cell division 1-like 1
192	2.42	ATG10	autophagy related 10
193	2.40	UQCRC2	ubiquinol-cytochrome c reductase core protein II
194	2.39	MED23	mediator complex subunit 23
195	2.32	COBL	cordon-bleu homolog (mouse)
196	2.29	YTHDC2	YTH domain containing 2
197	2.28	PTP4A1	protein tyrosine phosphatase type IVA, member 1
198	2.27	SLC39A9	solute carrier family 39 (zinc transporter), member 9
199	2.26	VGLL4	vestigial like 4 (Drosophila)

200	2.26	LAMA5	laminin, alpha 5
201	2.26	ATXN10	ataxin 10
202	2.22	PIK3CB	phosphoinositide-3-kinase, catalytic, beta polypeptide
203	2.22	IVNS1ABP	influenza virus NS1A binding protein
204	2.16	STX17	syntaxin 17
205	2.16	DNAJC1	DnaJ (Hsp40) homolog, subfamily C, member 1
206	2.14	SLC30A6	solute carrier family 30 (zinc transporter), member 6
207	2.08	RNF13	ring finger protein 13
208	2.08	VPS35	vacuolar protein sorting 35 homolog (S. cerevisiae)
209	2.06	ISOC1	isochorismatase domain containing 1
210	2.05	RNF34	ring finger protein 34, E3 ubiquitin protein ligase
211	2.03	WDR11	WD repeat domain 11
212	2.03	CEP63	centrosomal protein 63kDa

Supplementary Table S7 : Methylation data from the cell lines*

Gene	Expression status in RK	RK0	Other cell lines						Average other cell lines
			LoVo	LIM1215	KM12	HCT15	HCT116	HCA7	
**CMTM3**	up	**0.41**	0.88	0.91	0.94	0.90	0.84	0.99	**0.91**
**EpCAM**	down	**1.2**	0.13	0.11	0.14	0.11	0.14	0.13	**0.13**
**KLK10**	down	**0.49**	0.08	0.22	0.05	0.03	0.04	0.25	**0.11**
**DLC_i4**	up	**0.06**	0.82	0.53	0.05	0.05	0.85	0.10	**0.40**
**DLC1**		**0.03**	1.17	0.16	0.05	0.05	0.86	0.00	**0.38**

*some data were missing for the two genes DGKI and OPCML because of technical problems and unreliability
